# “Project for a Spatiotemporal Neuroscience” – Brain and Psyche Share Their Topography and Dynamic

**DOI:** 10.3389/fpsyg.2021.717402

**Published:** 2021-10-14

**Authors:** Georg Northoff, Andrea Scalabrini

**Affiliations:** ^1^Faculty of Medicine, Centre for Neural Dynamics, The Royal’s Institute of Mental Health Research, Brain and Mind Research Institute, University of Ottawa, Ottawa, ON, Canada; ^2^Mental Health Centre, Zhejiang University School of Medicine, Hangzhou, China; ^3^Centre for Cognition and Brain Disorders, Hangzhou Normal University, Hangzhou, China; ^4^Department of Psychological, Health and Territorial Sciences (DiSPuTer), D’Annunzio University of Chieti-Pescara, Chieti, Italy

**Keywords:** spatiotemporal neuroscience, neuropsychoanalysis, self, psychotherapy, spontaneous activity of the brain, common currency, brain and psyche

## Abstract

What kind of neuroscience does psychoanalysis require? At his time, Freud in his “Project for a Scientific Psychology” searched for a model of the brain that could relate to incorporate the psyche’s topography and dynamic. Current neuropsychoanalysis builds on specific functions as investigated in Affective and Cognitive (and Social) Neuroscience including embodied approaches. The brain’s various functions are often converged with prediction as operationalized in predictive coding (PC) and free energy principle (FEP) which, recently, have been conceived as core for a “New Project for Scientific Psychology.” We propose to search for a yet more comprehensive and holistic neuroscience that focuses primarily on its topography and dynamic analogous to Freud’s model of the psyche. This leads us to what we describe as “Spatiotemporal Neuroscience” that focuses on the spatial topography and temporal dynamic of the brain’s neural activity including how they shape affective, cognitive, and social functions including PC and FEP (*first part*). That is illustrated by the temporally and spatially nested neural hierarchy of the self in the brain’s neural activity (*second and third part*). This sets the ground for developing our proposed “Project for a Spatiotemporal Neuroscience,” which complements and extends both Freud’s and Solms’ projects (*fourth part*) and also carries major practical implications as it lays the ground for a novel form of neuroscientifically informed psychotherapy, namely, “Spatiotemporal Psychotherapy.” In conclusion, “Spatiotemporal Neuroscience” provides an intimate link of brain and psyche by showing topography and dynamic as their shared features, that is, “common currency.”

“Every attempt to discover a localization of mental processes … has miscarried completely. The same fate would await any theory that attempted to recognize the anatomical position of the system (consciousness) – as being in the cortex, and to localize the unconscious processes in the subcortical parts of the brain. There is a hiatus which at present cannot be filled, nor is it one of the tasks of psychology to fill it. Our psychical topography has for the present nothing to do with anatomy.” ~Freud, 1915.

## Introduction

How can we link psychoanalysis to neuroscience? Freud himself tried to connect psychoanalysis and neuroscience in his early writing “Project of a Scientific Psychology” (1895). However, in the following, he gave up on such project focusing mainly on the development of psychoanalysis. These efforts flare up again our time including various clusters of recent neuroscientific research (Boeker, 2018). One cluster is the “*embodied brain hypothesis”* that conceives cognitive and affective functions of the brain to be closely linked to the body and its interoceptive functions. This has led to concepts like “*embodied remembering*,” “*embodied unconscious*,” “*embodied memories*,” “*embodied feelings*,” and “*embodied testimony*” (Edelman, 1987, 1989, 1992; Northoff, 2011, 2012a; Leuzinger-Bohleber, 2018; Mucci, 2019).

Yet another cluster of neuroscientific research is the dynamic neuropsychology by AR. Luria that, through Solms’ clinical-anatomical localization approach to psychodynamic features, provides one link of brain and psyche. Moreover, in addition to Cognitive Neuroscience, the development of Affective Neuroscience by especially Jaak Panksepp (1998) represents another cluster where primary and secondary emotions are linked to primary and secondary processes (Panksepp and Biven, 2012; Solms, 2015). Finally, yet another more recent cluster is developed by Mark Solms when he aims to link the free energy and predictive coding approach by Karl Friston to psychoanalysis: He considers the biologically and physically defined concepts of free energy and predictive coding to reflect what Freud referred to as mental or psychical energy (Solms and Friston, 2018; Solms, 2020, 2021). That, according to Solms, provides the key connection of brain and psyche as core feature of a “New Project for a Scientific Psychology” (Solms, 2020, 2021).

Despite all progress, neuroscientific approaches adhere to a scientific psychology that, as traditionally conceived, is based on specific functions and the third-person perspective. Just as the psyche in psychology, the brain is conceived in terms of specific functions showing extrinsic contents, affective, cognitive, or social: these are localized in particular regions of the brain, remain the same over time and are investigated by probing the brain’s task-related activity. As in the case of the psyche in psychology, this amounts largely to a non-energetic, mostly static, content-based, and third-person-based view of the brain.

**Figure 1:**
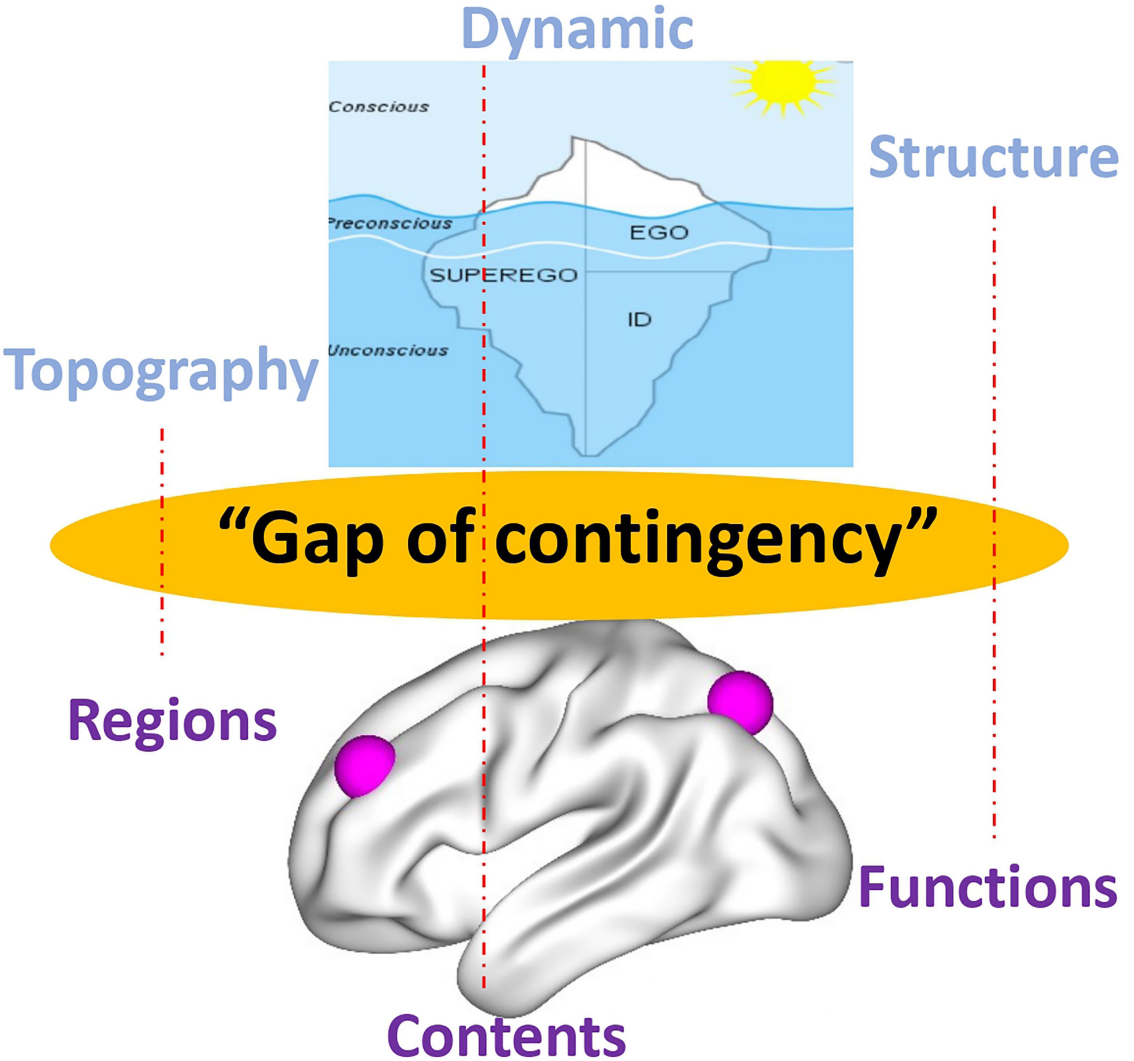
Different models of the psyche in psychoanalysis (upper) and the brain in current neuroscience (lower) with red dotted lines indicating insufficient contingent connection, that is, “gap of contingency.”

**Figure 2:**
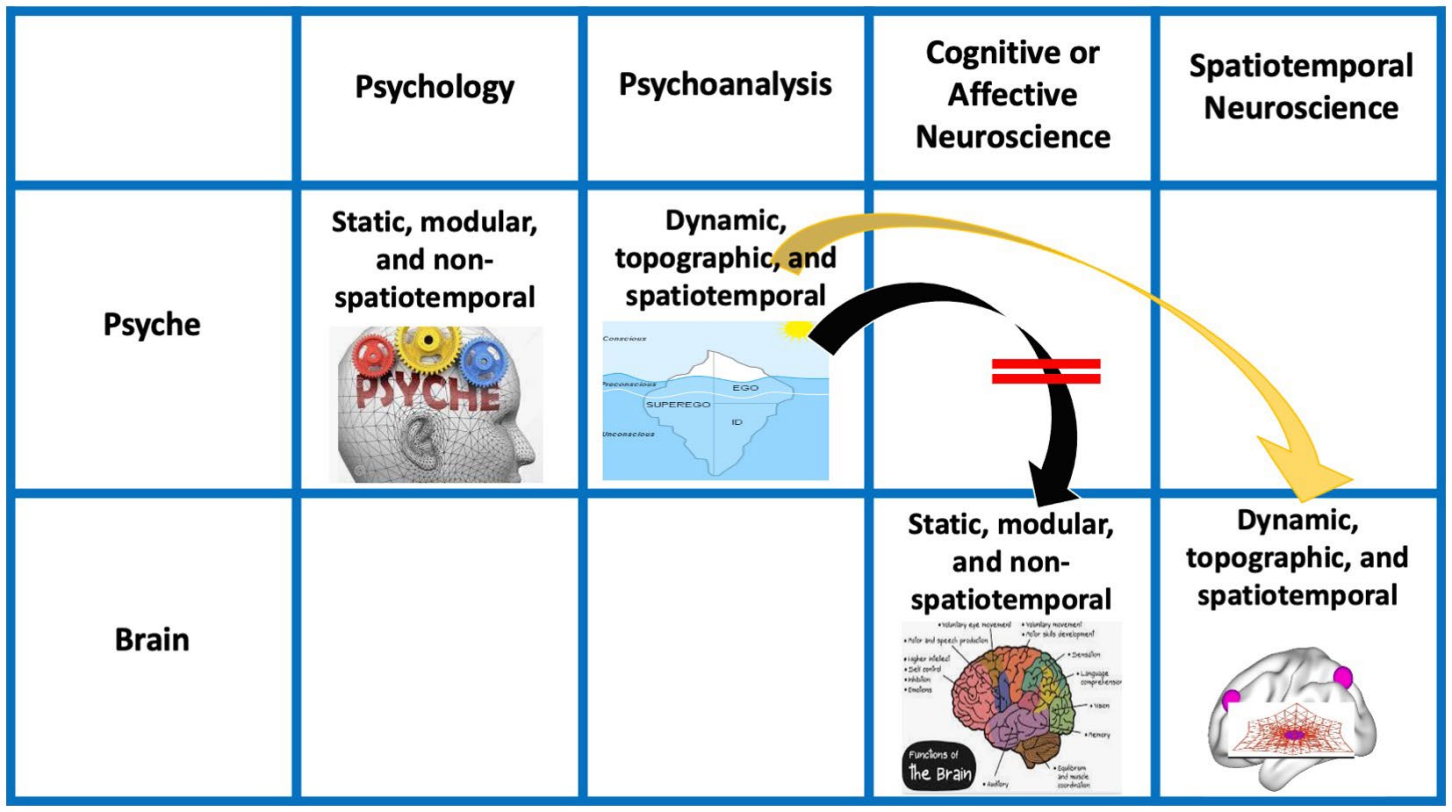
Views of psyche and brain in psychology, psychoanalysis, and different forms of neuroscience. Arrows indicate the combination of the models of the psyche in Psychology and Psychoanalysis with the model of brain as in Cognitive/Affective Neuroscience (green arrow, black arrow) as well as of psychoanalysis with Spatiotemporal Neuroscience (orrange arrow). Red lines (on the black arrow) indicate the mismatch between the models of psyche (in psychoanalysis) and brain (in Coginitive/Affective Neurosscience).

Psychoanalysis, in both its original inception and current manifestations, contends such view of the psyche as presupposed in psychology. Instead, the psyche is conceived as highly energetic (like cathexis) rather than non-energetic, it is continuously changing, and thus, dynamic rather than static exhibits a structure or organization that shapes its contents and aims for a first- or second- rather than third-person-based view of the psyche (e.g., Northoff et al., 2007; Schilbach, 2010; Przyrembel et al., 2012; Longo and Tsakiris, 2013). This leaves a gap, a “gap of contingency” (*see below*), when compared to the current view of the brain in neuroscience. The energetic, dynamic, structural/organizational, and first/s-person features of the psyche in psychoanalysis are related to a brain that is non-energetic, static, content-based, and third-person-based. Such mismatch between the models of psyche (in psychoanalysis) and brain (in affective, cognitive, and social neuroscience) renders impossible to take into view their intimate connection, that is, how neural activity transforms into psychic activity. Our view of brain-psyche is consequently blocked by a gap with their connection remaining contingent (rather than necessary *a posteriori*; Northoff, 2018) – we will therefore speak later of a “gap of contingency.” (see the dotted lines and black lines in Figure 1 and the black arrow in Figure 2 as below).

How can we close the “gap of contingency” between brain and psyche? One way is to take into view the brain in terms that are analogous to the model of the psyche in psychoanalysis. Specifically, one may want to conceive the brain in terms of its energy, dynamic, structure/topography, and first/s-person perspective. Brain and psyche can then be conceived in analogous terms with the ultimate hope that these features are shared by brain and psyche as their “common currency” (Northoff et al., 2020a). Importantly, that should close the current “gap of contingency” between brain and psyche allowing for their tighter connection (i.e., necessary *a posteriori*; Northoff, 2018) as searched for by both Freud and Solms in their respective projects for a scientific psychology.

The goal of our paper is to develop the kind of neuroscience that is necessary to intimately connect brain and psyche in order to complete Freud’s “Project for a Scientific Psychology” (1895). Rather than relying on the characterization of the brain as in current Cognitive and Affective and Social Neuroscience, we propose taking an alternative view, one that focuses on the brain’s energy, dynamic, structure, and conceives it in first/second-person perspective. Specifically, we aim to develop a more comprehensive and holistic model of the brain in terms of its intrinsic temporal dynamic and spatial topography – this requires “Spatiotemporal Neuroscience” (Northoff et al., 2020a,b; *first part*). That approach will be illustrated empirically by the example of the self featured by its topography (*second part*) and dynamic (*third part*) in brain and psyche.

Spatiotemporal Neuroscience carries major theoretical implications for the “Project for a Scientific Psychology” by both Freud (1895) and Solms (2020). Additionally, it carries major practical implications as it lays the ground for a novel form of neuroscientifically informed psychotherapy, namely “Spatiotemporal Psychotherapy” (*fourth part*). We conclude that “Spatiotemporal Neuroscience” provides a strong candidate for complementing Freud’s unfinished “Project for a Scientific Psychology” (1895) including its most recent version of a “(New) Project for a Scientific Psychology” by Mark Solms (2020, 2021). We therefore speak of a “Project for a Spatiotemporal Neuroscience.”

## Part I: Psychoanalysis and Neuroscience – Views of Psyche and Brain

### Psyche in Psychoanalysis – Dynamic, Topographic, and Spatiotemporal

One of Freud’s key observations was that the psyche is dynamic, that is, it changes over time with the changes following a certain pattern that establish a particular structure or organization. The emphasis on the dynamics of the psyche is well reflected in his notion of mental or psychic energy, that is, cathexis, that fuels drives, libido, instinct, and the dynamic unconscious where cathexis remains unconstrained. This mental energy is key in structuring and organizing the psyche in a dynamic way. That is reflected in his first topographical model of the unconscious-conscious as well as in his second topographical model of the three-fold relation of *Id*, *Ego*, and *Super-ego*.

Freud aimed to decipher a deeper and more fundamental layer of the psyche beneath its functions and contents when focusing on its dynamic and topography. How, though, can we describe dynamic and topography of the psyche independent and prior of their functions and contents? Freud himself emphasizes the spatial and temporal features of the psyche – we may require a spatiotemporal approach complementing the affective and/or cognitive approach to the psyche. This is well reflected in the following quote by Freud himself.

*“Accordingly, we will picture the mental apparatus as a compound instrument, to the components of which we will give the name of ‘agencies’, or (for the sake of greater clarity) ‘systems’. It is to be anticipated, in the next place, that these systems may perhaps stand in a regular **spatial** relation to one another, in the same kind of way in which the various systems of lenses in a telescope are arranged behind one another. Strictly speaking, there is no need for the hypothesis that the psychical systems are actually arranged in a spatial order. It would be sufficient if a fixed order were established by the fact that in a given psychical process the excitation passes through the systems in a particular **temporal** sequence. In other processes the sequence may perhaps be a different one; that is a possibility that we shall leave open. For the sake of brevity we will in future speak of the components of the apparatus as ‘ψ-systems’”* (Freud 1900, p. 535; *bolds by us*).

### Psyche in Psychology – Static, Modular, and Non-spatiotemporal

The conception of the psyche in psychoanalysis by dynamic, topography, and spatiotemporal features stands in contrast to the view of the static view of the psyche. The psyche is often conceived as collection of functions in terms of modules that are merely added together, standing side-by-side in parallel. For instance, different memory systems (like working memory, semantic, and episodic memory) and distinct emotions or form of attention are distinguished from each other operating more or less independently or in a modular way. Accordingly, there is no assumption of an overall psychic structure, that is, topography encompassing all functions in an organized whole.

Moreover, the various psychological functions are considered to be stable and non-changing thus being static – the dynamic beneath the functions is thus often neglected. Together, this amounts to a view of the psyche in psychology in terms of functions and their contents while their underlying spatial and temporal features are neglected. This stands in contrast to the view of the psyche in psychoanalysis where spatial and temporal features are assumed to shape and constitute the psyche (*see above*).

Finally, there is also a methodological difference between psychoanalysis and psychology regarding first/second vs. third-person perspective. Psychoanalysis requires first/second person reports with subjective experience for understanding the dynamic manifestations of psychic energy as well as the structure of conscious-unconscious and *Id–Ego–Super-Ego*, that is, their topography. That stands in contrast to psychology. Here, the focus is on objective observation in third-person perspective as to eclipse and exclude any traces of subjective first/second perspective. Even stronger, first/second person experience is often criticized as non-scientific in conventional psychology that focuses strictly on third-person perspective to acquire data. Hence, the dynamic vs. static approach to the psyche stand in opposition and are exclusive on methodological grounds. This makes urgent a more cohomprensive and holistic approach.

### View of the Brain in Cognitive, Affective, and Social Neuroscience – Static, Regional Modular, and Non-spatiotemporal

Neuroscience and its different branches like cognitive, affective, social, and cultural (just to name a few) are developed largely as extension of the respective branches in psychology. This means that the static, modular, non-spatial and non-temporal, and third-person-based view of the psyche is more or less transferred to the brain itself.

Particular cognitive or affective functions are associated with specific brain regions whose neural activity, as related to these functions, is conceived non-changing, that is, static, and modular, that is, localized in specific brain regions (like the localization of primary and secondary emotions in distinct subcortical and cortical regions; Panksepp, 1998). The brain itself and its neural activity are consequently conceived as static, modular, non-spatiotemporal, and third-person-based. This view of the brain, although necessary for the discover of brain-related functions and contents, predominates in current Cognitive and Affective Neuroscience (and related branches like Social and Cultural Neuroscience) and lacks of a more holistic and comprehensive approach.

The primacy of functions and contents goes along with a focus on task-related activity that measures the impact of the former on the brain’s neural activity. Analogously to the functions themselves, task-related activity is then also considered in a static and regional modular way independent of potentially underlying spatial and temporal features. Taken together, Cognitive and Affective Neuroscience (and their various siblings) considers the brain and its task-related activity in more or less the same terms as the psyche is viewed in psychology.

Given such analogy in their characterization, the brain and their task-related activity are supposed to account for the psyche thus bridging the gap between neuroscience and psychology (see green arrow in Figure 2). In contrast, such view of the brain does not bridge the gap to the view of the psyche in psychoanalysis as that is dynamic, topographic, and spatiotemporal rather than static, modular, and non-spatiotemporal (see black arrow with red bars in Figure 2).

### “Common Currency” – Temporal Dynamic and Spatial Topography Are Shared by Brain and Psyche

How can Cognitive, Social, and Affective Neuroscience account for the psychodynamic view of the psyche? The various clusters of their connection pointed out in the introduction cannot but suffer from a fundamental discrepancy between brain and psyche. They all aim to connect a static, regional modular, and non-spatiotemporal brain, featured by its extrinsic task-related activity, with a dynamic, topographic, and spatiotemporal psyche characterized by its intrinsic spontaneity. The only way to remedy this discrepancy is to view the brain in a way that is analogous to the psyche in psychoanalysis. The brain and its neural activity may thus need to be conceived as dynamic, topographic, and spatiotemporal – this is the aim of what recently has been introduced as “Spatiotemporal Neuroscience” (Northoff et al., 2020a,b).

One key feature of the brain is its spontaneous activity that refers to the absence of specific tasks or stimuli as it can be measured during the resting state (Logothetis et al., 2009; Raichle, 2009, 2010; Northoff, 2012b, 2014a,b, 2018). The spontaneous activity can be characterized topographically by various interacting networks like default-mode network, salience network, and central executive network, whose relationships seem to be modulated by the brain’s global activity, that is, global signal topography (Tanabe et al., 2020; Zhang et al., 2020; Scalabrini et al., 2020a). While on the temporal side, the brain’s spontaneous activity is characterized by fluctuations or oscillations in various frequency ranges (*see below for details*) that, together, provide a certain temporal dynamic structure (Buzsaki, 2006; He, 2014; Scalabrini et al., 2017; Northoff, 2018; Scalabrini et al., 2019).

The spontaneous activity itself has recently been associated with various internally oriented cognitive functions like mind-wandering (Smallwood and Schooler, 2015; Christoff et al., 2016; Northoff, 2018), mental time travel or episodic simulation (Schacter et al., 2012; Northoff, 2017), autobiographical memory, and self-referential processing (Northoff et al., 2006; Northoff, 2016). Hence, the spatial topography of the spontaneous activity may itself be related to different forms of cognition (Smallwood et al., 2021; Yeshurun et al., 2021). This leaves open how the spontaneous activity mediates such cognitive (and also affective and social) functions during both resting state and task-related activity, though. Addressing this question is key in providing an intimate connection of brain and cognition/emotion, that is, of neural and psychological activity and hence of brain and psyche.

We postulate that, in order to provide such intimate connection, brain and psyche must share some features, a “common currency” (Northoff et al., 2020a,b). Freud’s topography and dynamic of the psyche entails a spatial and temporal view of the psyche: the temporal and spatial organization and structure of the psyche shapes its contents and functions. Relying on Freud and our spatiotemporal characterization of the brain’s spontaneous activity, we now propose that spatial topography and temporal dynamic are shared by both neural and psychical activity. What Freud described as mental topography and dynamic of the psyche characterizes also, in more or less analogous ways, the brain’s neural activity including both spontaneous and task-related activity. Spatial topography and temporal dynamic are thus shared as “common currency” of brain and psyche (Northoff et al., 2020a,b).

### View of the Brain in Spatiotemporal Neuroscience – Dynamic, Topographic, and Spatiotemporal

We are now ready to determine what we recently introduced as “Spatiotemporal Neuroscience” (Northoff et al., 2020a,b). As explicated above, Cognitive, Affective, Social and Cultural Neuroscience largely view the brain as static, regional modular, and non-spatiotemporal. This contrasts with Spatiotemporal Neuroscience that conceives the brain’s neural activity (including both spontaneous and task-related activity) largely in dynamic, topographic, and spatiotemporal terms.

**Figure 3:**
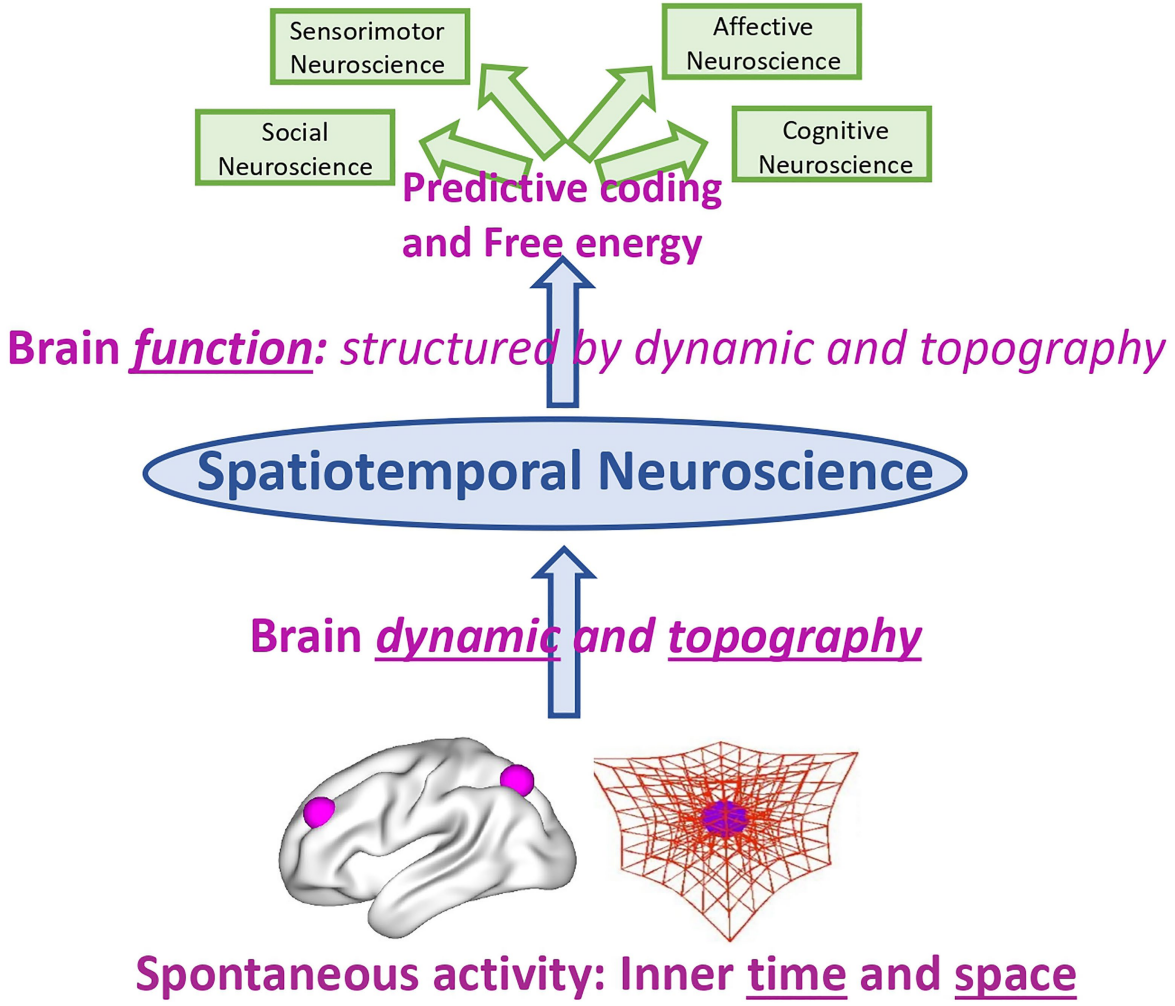
Spatiotemporal Neuroscience – from brain dynamic and topography to brain function.

Rather than on the neural activity of affective, cognitive, etc. functions and contents themselves, the focus in Spatiotemporal Neuroscience is on the spatial topography and temporal dynamic of their neural activity during both internally and externally oriented cognition. Spatiotemporal Neuroscience thus conceives both the brain’s neural activity and the psyche’s mental activity primarily in spatial topographic and temporal dynamic terms: It focuses on the brain’s spatial and temporal features that constitute its dynamic and topography, and how they, in turn, shape cognitive, affective, and social brain function including their respective contents. This makes it clear that Spatiotemporal Neuroscience neither stands contradictory to nor is exclusive with Affective, Cognitive, and Social Neuroscience. Instead, the former integrates and embeds the letter in a broader more comprehensive spatial and temporal context, that is, topography and dynamic (see Figure 3).

The same also applies to predictive coding and free energy. Spatiotemporal Neuroscience provides the spatial topographic and temporal dynamic context within which predictive coding and free energy operate; they provide what has been called “deep temporal model” (Kiebel et al., 2008; Friston et al., 2017) or “temporal thickness” (Seth, 2015). Spatiotemporal Neuroscience focuses primarily on the dynamic and topographical features that for instance characterize free energy (Friston et al., 2015), that indeed has been demonstrated to be scale-free and operates at multiple nested spatial scale and timescale. The same applies to the link of first and third person. Spatiotemporal Neuroscience takes into consideration first-person experience of mental features and links them to third-person observation about the brain – this link is made possible through spatiotemporal features being shared by both first and third person as their “common currency.” This shall now be demonstrated by the example of self whose topography (*second part*) and dynamic (*third*) in both its psyche and brain are discussed in the next two parts.

## Part Ii: Spatial Topography – Nestedness As “Common Currency” of Self and Brain

### Topography of the Brain – Spatial Layers and Nested Hierarchy

Topography refers to a particular spatial organization or structure of brain and psyche. One key feature of their topography is hierarchies. Hierarchies have been postulated in both neuroscience and psychoanalysis. They may thus offer insight into the intimate connection of, for instance, brain and self. The English neurologist Hughling Jackson early on proposed a three-layer hierarchy of the brain with lower, middle, and higher centers that were assumed to be associated with different regions and psychological functions (see Wiest, 2012 for an overview). More recently, MacLean (1990) and Panksepp (1998, 2012) conceived the brain’s subcortical–cortical organization in terms of a radial-concentric pattern and associated its different layers different levels of emotions (like primary, secondary, and tertiary emotions). Panksepp (1998) and Damasio (2010) associated such hierarchy of the brain with different concepts of self, like bodily self, autobiographical self, and extended self. A more radial-concentric approach to the brain is the three-layer anatomical model of the brain as proposed by Feinberg and Northoff (Northoff et al., 2011). We will see that, together with the recent data, this supports the idea of a spatially nested hierarchy of self based on the brain’s radial-concentric organization.

A clearly hierarchical organization of self (which we here use in a broader sense) which embeds and contains the concept of ego has also been proposed in psychoanalysis by Freud. Strachey (1961) and Kernberg (1984) noted how Freud preserved the German *Ich – Ego* as a mental structure and psychic agency but also as the subjective experiential self in all his writing. In synthesis, Strachey and Kernberg propose that Freud never dissociated the *Ich – Ego* from the experiencing self. Moreover, Freud suggested the *Id* to be the lowest level of the topography of the psyche that remains essentially unconscious but nevertheless strongly influences the upper levels of the *Ego* and the *Super-ego*.

Together, this amounts to a nested hierarchy of self where the lower layer somewhat re-surfaces within the next upper layer and so forth (see also Wiest, 2012). While Freud’s rigid three-layer partition was criticized later by others, the multi-facedness of self with its sense of subjectivity permeating across bodily, affective, and cognitive layers remains a key feature in both neuroscience and psychoanalysis. We will demonstrate that the model of a nested hierarchy of self is strongly supported by recent neuroscience in both its spatial and temporal aspects – therefore, we characterize the neural (and psychological) hierarchy of self by spatial and temporal nestedness.

### From the Brain’s Topography to the Self – Three Input Layers (Interoceptive, Extero-Proprioceptive, Mental)

**Figure 4:**
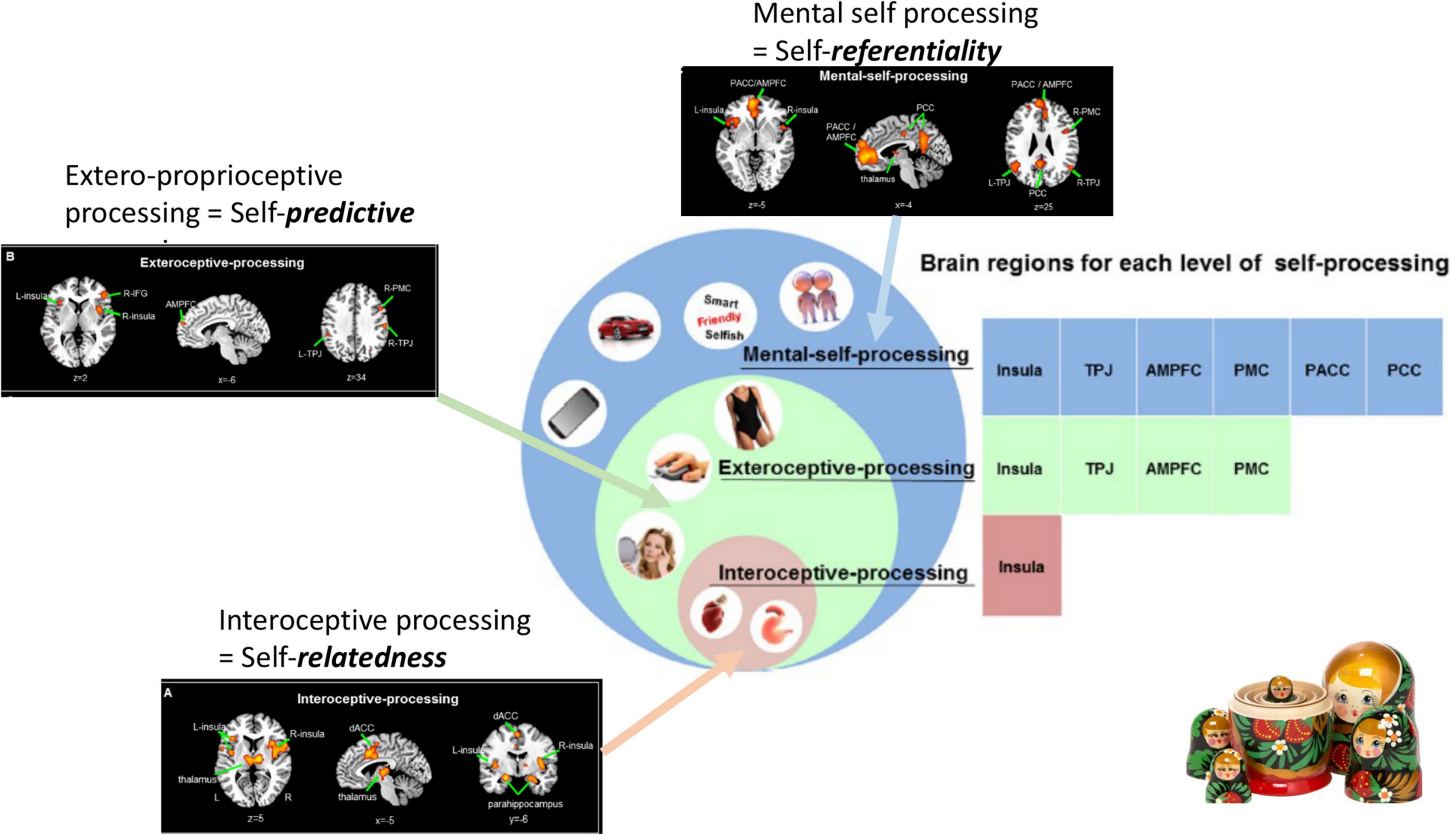
Nested hierarchy of self in the brain with different regional layers of brain activity (black boxes on the left and upper middle) nesting in their regions within each other (brown, green and blue boxes on the right) resulting in a corresponding nestedness of different layers of self (brown, green, and blue circles in the middle).

A recent large-scale meta-analysis in healthy subjects by Qin et al. (2020) investigated and analyzed different imaging studies that focused on different aspects of self, inner body (interoceptive), outer body (extero-proprioceptive), and the own cognitive or mental states. They observed different regions to be associated with each of the three layers; at the same time, there was regional overlap as the regions of the lower layer were included within the next upper layer (*see below for details*). Together, this amounts to a spatial multi-layered nested hierarchical model of self (Qin et al., 2020) including (1) interoceptive self (2) extero-proprioceptive self, and (3) mental self (Figure 4).

The interoceptive self, that is, how the brain processes and perceive the body’s inner organs and their input, was investigated through fMRI task studies that measured interoceptive awareness of the own body including cardiorespiratory awareness, urogenital, and gastrointestinal awareness. That was complemented by extero-proprioceptive self fMRI studies focusing on external bodily inputs like facial or proprioceptive inputs connected to the self. Finally, they also included the “typical” more cognitive mental self fMRI studies employing trait adjectives or other stimuli where subjects have to become aware of their own self as distinct from others.

Based on the interoceptive studies, there is a most basic or lower layer of self, an interoceptive self that is related to regions that mostly process interoceptive stimuli from the own body, that is, bilateral insula, dorsal anterior cingulate cortex, thalamus, and parahippocampus thus including mainly regions of the salience network (Menon, 2011; Qin et al., 2020). The fact that these regions were shared among the different kinds of interoceptive awareness, that is, cardiorespiratory, urogenital, and gastrointestinal, suggests that these regions are key in integrating different interoceptive inputs of the various organs of the inner body (Craig, 2003, 2010). One can thus speak of an *“interoceptive or vegetative self”* (Babo-Rebelo et al., 2016, 2019), “*bodily self*” (Tsakiris, 2017), “*proto-self*” (Damasio, 2010), or “*SELF*” (Panksepp, 1998) as most basic and fundamental layer of self. Following particularly Panksepp (1998), the SELF provides a complex network infrastructure where all basic emotional operating systems converge on primitive brain regions such as the thalamus and the periaqueductal gray (PAG). This network is similar across all mammalian species and represents the interoceptive and thus affective foundation of SELF and necessary for the construction of higher levels of self (Northoff and Panksepp, 2008). This is in line with the interoceptive level of self-processing proposed by Qin et al. (2020) that is considered the ground level of the hierarchy.

The next or middle layer of self includes what Qin et al. (2020) describe as proprioceptive or exteroceptive self; the fMRI studies focusing on external bodily-related inputs like facial or proprioceptive inputs yielded regions like bilateral insula, interior frontal gyrus, premotor cortex, temporo-parietal junction (TPJ), and medial prefrontal cortex. As these regions process inputs from different sensory modalities, they may be key in not only integrating extero- and proprioceptive modalities but also different exteroceptive sensory modalities, that is, cross-modal integration. Despite their differences, these regions share the processing of proprioceptive inputs related to the own body – one can thus speak of a “*proprio- or extero-ceptive self, or embodied self”* (Panksepp, 1998; Gallagher, 2005; Damasio, 2010; Tsakiris, 2017).

Finally, the most upper layer of self (Qin et al., 2020) is based on fMRI studies that yielded typical DMN midline regions like medial prefrontal cortex and posterior cingulate cortex as well as the regions included in the second level, most notably bilateral TPJ, and first level, bilateral insula and thalamus. These regions seem to be recruited when on needs represent one’s own self in mental states – one can therefore also speak of a *“mental or cognitive self”* (Qin et al., 2020) or “*extended self*” (Damasio, 2010).

Together, these findings suggest what Qin et al. (2020) describe as “nested hierarchy of self”: Regions of the lower level were included in the next higher level where they were complemented by additional regions and so forth. For instance, bilateral insula was present on the most basic level, that is, the interoceptive self and resurfaced (in completely independent imaging studies) again in both second, that is, proprio-exteroceptive, and third, that is, mental self, levels. The same hold true for the bilateral TPJ that first showed in the intermediate layer of the proprioceptive self and re-resurfaced again in the third level of the mental self. Accordingly, each of the hierarchical levels of self recruits both overlapping and separate regions compared to other levels amounting to spatial nestedness with a spatially nested hierarchy of self (Figure 4).

### Spatial Topography of Self – Tripartite Structure of Ego Vs. Different Input Layers of Self

**Figure 5:**
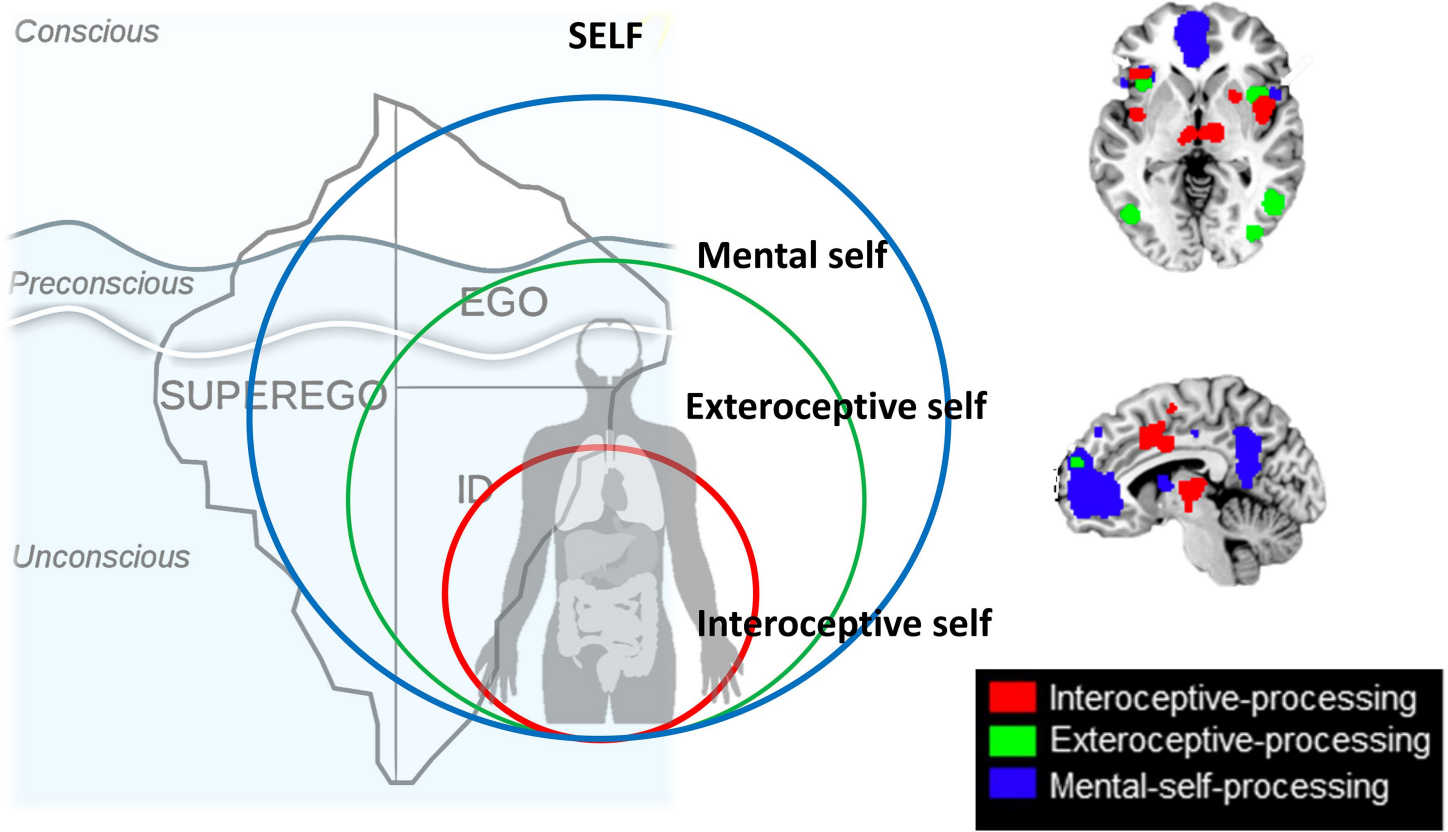
The model of self that embeds and contains the topographical model of Id-Ego-Superego conceptualized by Freud.

The constitution of the nested hierarchy of self by the brain provides close connection to psychoanalysis. We here refrain from associating the spatial topographical findings of the brain with the tripartite psychodynamic topography proposed by Freud (since it might be rather too speculative and unprecise; see Figure 5). Instead, we pursue another more empirical path where the empirical data are converged with a more relational model of self.

We have seen that all three layers of self, interoceptive, proprio-exteroceptive, and cognitive mental, are based on the respective inputs from the inner body, the outer body, and the cognitive (and ultimately neuronal) input from within the brain itself. The self, operationalized as a whole subjective experience at different nested levels and different from the ego described by Freud, is here operationalized in terms of its input processing, that is, how it processes and relates to the distinct types of inputs, that is, interoceptive, proprio-exteroceptive, and cognitive mental.

Importantly, the self is here no longer conceived conceived as an isolated entity that “resides” inside the inner regions and structures of brain, body, and mind. Instead, the self is constituted by processes that reach beyond the boundaries of brain, body, and mind to the external world by taking on different degrees of expansion, that is, self-expansion (Northoff, 2016; Scalabrini et al., 2018).

First, the self is constituted by integrating the different interoceptive inputs from within the inner body – the interoceptive self. Second, these processes expand beyond the inner body by reaching out to the outer body, the proprio- or exteroceptive self. Third, these processes extend beyond the outer body to the brain and its cognitive input – this is the mental or cognitive self. While targeting distinct inputs, these processes constitute a sense of self that reaches beyond the boundaries of brain, body, and environment: They, as we will lay out below, constitute a virtual three-dimensional spatial structure that integrates brain, body, and environment by nesting them within each other, that is, spatial nestedness.

### Topography of Brain and Psyche – Spatial Nestedness as “Common Currency” of Brain and Self

We take the self as paradigmatic instance about the relation of brain and psyche. Specifically, we demonstrate how the topography of the brain constitutes a particular spatial structure or organization across all of its regions/networks. This suggests the importance of spatial topography for the brain at a deeper and more fundamental layer, that we intend as a deeper organizational and structuring principle, beyond its single regions/networks with their respective cognitive, affective, and social functions.

Even more relevant, we could demonstrate how the brain’s topographic organization of spatial nestedness is related to correspondingly nested layers of self. This supports, albeit tentatively, the assumption that spatial topography, that is, spatial nestedness is shared as “common currency” by both brain and self and, more generally, by brain and psyche.

Freud and psychoanalysis target a deeper layer of the mind. Rather than focusing on the conscious at the surface, they venture into the unconscious depth of our psyche. The view of the brain in terms of topography (and dynamic) now allows to take into view a corresponding depth layer within the brain itself. Rather than on specific functions with their affective, cognitive, or social contents (either conscious or unconscious), the focus is here on topography (and dynamic), something that eludes even our unconscious let alone our consciousness – it is the non-conscious brain that yields our unconscious psyche.

We see here that such most fundamental or depth layer of the brain is key in providing the structure or organization of the basic layers of the psyche like the spatial nestedness constituting the hierarchy of self. We consequently speak of a “Basis model of self-specificity” (BMSS) which, in a nutshell, states that self-specificity permeates all layers of input processing including interoceptive, exteroceptive, proprioceptive, and cognitive/mental (Northoff, 2016).

The Qin et al. (2020) study illustrates that such basic or fundamental sense of self with (Scalabrini et al., 2021) its different layers is featured by a particular topographic organization, that is, spatial nestedness as shared by both brain and self as their “common currency” (Northoff et al., 2020b). Such more basic and fundamental view of the role of self aligns more or less well with the various psychodynamic conceptions departing from an intra-psychic vantage point (e.g., Freud) and moving further to a more interpsychic or relational point of view of self as proposed by Kohut, Winnicott, Stern, Bromberg, Fonagy, Solms, and Panksepp and Biven (and many others; see Northoff, 2011; Scalabrini et al., 2018; as well as Spagnolo and Northoff, 2021 for details).

## Part Iii: Temporal Dynamic – Scale-Freeness As “Common Currency” of Brain and Self

*“The brain might be a transformer station, in which the relatively infinite tension or intensity of the psyche proper is transformed into perceptible frequencies or “extensions.”*~Carl Jung, Letters Vol. II, Pages 43–47.

### Dynamic of the Brain –Operation Across Different Timescales in a Scale-Free Way

The brain’s spontaneous neural activity can be characterized by different frequencies ranging from infraslow (0.01–0.1Hz), over slow (0.1 – 1hz), fast (1 – 40Hz) to ultrafast (40-180Hz; Buzsaki, 2006). Power is strongest in the infraslow range and decreases across the slow, fast, and ultrafast ranges following a power law distribution (He et al., 2010; He, 2014; Huang et al., 2016). Together, the different frequencies and their distinct degrees of power constitute a complex temporal structure in the brain’s spontaneous activity which, in large parts, can be featured by the balance between infraslow, slow, and faster frequencies.

**Figure 6:**
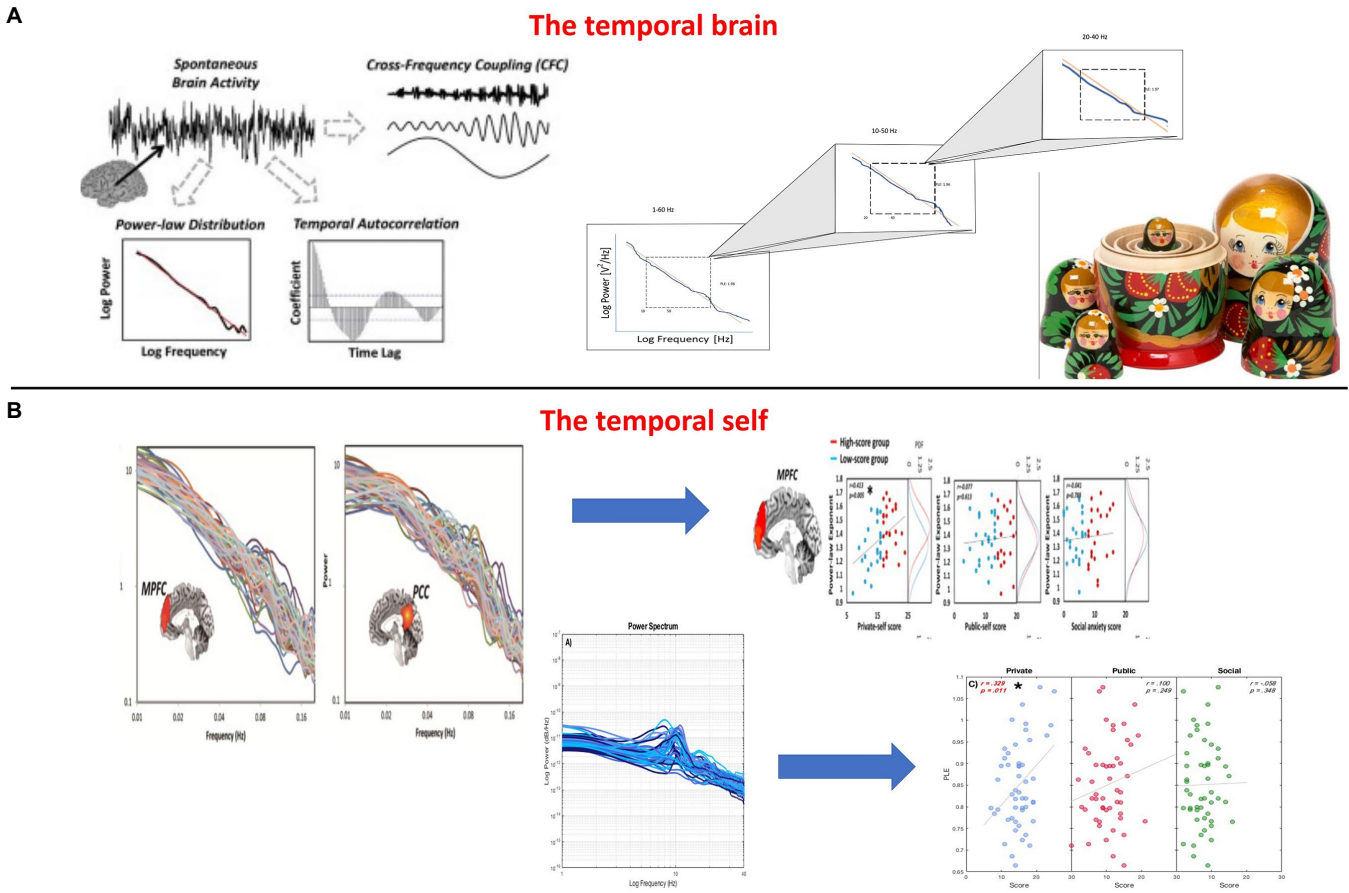
**(A)** The temporal brain – Temporal nestedness with scale-free activity in the brain (left and upper right) just like Russian dolls with their spatial nestedness (lower part). **(B)** The temporal self – From the brain’s scale-free activity with its temporal nestedness to the self in infraslow frequencies of fMRI (upper right/Huang et al., 2016) and faster frequencies of EEG (lower right, Wolff et al., 2019).

The relationship between these frequencies is maintained across different temporal scales and can therefore be characterized by what is described as “scale-free dynamics” (Linkenkaer-Hansen et al., 2001; He et al., 2010; He, 2014). Roughly, scale-free activity describes the fractal (i.e., self-similar) organization and thus temporal nestedness in the relationship between power and the different frequency ranges: the longer and more powerful slower frequencies nest and contain the shorter and less powerful faster frequencies – this amounts to long-range temporal correlation (LRTC) which operates across different time scales or frequencies (Linkenkaer-Hansen et al., 2001; He et al., 2010; He, 2014; Northoff and Huang, 2017; see Figures 6A,B).

The LRTC makes it possible to assess the degree to which past neuronal patterns exert their influence on future dynamics, thus accounting for LRTC (Linkenkaer-Hansen et al., 2001; Northoff and Huang, 2017). That amounts to a form of memory that is here defined not by specific contents that are encoded, stored, and recalled or retrieved. Instead, memory refers here to the structure, the temporal structure of the neural activity across distinct time points. One could thus speak of temporal memory or dynamic memory, that is, process- and structure-based memory, as distinct from the more content-based cognitive memory in the traditional sense (Hasson et al., 2015; Northoff, 2017). Accordingly, LRTC and henceforth scale-free activity provide not only temporal stability through their correlation of different timescales, that is, temporal continuity, but also temporal memory, that is, temporal stability, through connecting past, future, and present timepoint (Figure 6A).

### The Scale-Free Self – The Brain’s LRTC Shape the Self

Is the self related to the LRTC of the brain’s neural activity? Recent studies have shown that the brain’s scale-free activity, as measured with either Power Law Exponent (PLE) or Detrended Fluctuation Analysis, is related to mental features such as the self (Huang et al., 2016; Scalabrini et al., 2017, 2019; Wolff et al., 2019). Together, these studies show that the degree of resting state PLE directly predicts: (1) the degree of self-consciousness (Huang et al., 2016; Wolff et al., 2019) (2) task-related activity during self-specific stimuli (Scalabrini et al., 2019), and (3) the degree of temporal integration on a psychological level of self-specificity (Kolvoort et al., 2020).

Let us describe the findings in more detail. Huang et al. (2016) and Wolff et al. (2019) recorded resting state activity in fMRI and EEG of the brain, that is, a task-free condition without any external demands. They calculated the degree of the brain’s PLE in both fMRI and EEG. The same subjects also underwent psychological investigation of their self with the self-consciousness scale. Both studies found the same relationship of brain PLE and self-consciousness: The higher the PLE, that is, the more the slow-fast power balance is shifted toward the slow pole, the higher the degree of the subject’s private self-consciousness (see Figure 6B).

Importantly, these findings hold only for the PLE as index of slow-fast balance but not for either the slow or fast frequencies alone. Finally, it shall be mentioned that this concerns a wide range of frequency range, from very slow (0.01 to 0.1Hz), as covered by fMRI (Huang et al., 2016), to faster ones as measured in EEG (1-80Hz) (Wolff et al., 2019). This means that it is the degree slow-fast integration, that is, their degree of scale-freeness, that is related to the sense of self. The self is thus intrinsically scale-free as it connects and links different timescales short/fast and long/slow. Such cross-scale self exhibits both temporal continuity and discontinuity and nests them within each other in a scale-free way: temporal continuity, as mediated by the more powerful slower frequencies, nests and contains temporal discontinuity, as related to the less powerful faster frequencies.

### Dynamic of Brain and Psyche – Scale-Freeness as “Common Currency” of Brain and Self

Is such self-specificity of the brain’s internal resting state activity also carried over to external task demands during self-specific tasks? This was studied in fMRI by Scalabrini et al. (2017, 2019). He measured both rest and task during the active touch toward an animate (another person) and non-animate (mannequin hand) targets. They observed that the degree of PLE in the resting state predicted the degree to which subjects could differentiate in their task-related activity between animate and non-animate targets. Given that rest and task states occur and are measured at distinct points in time, this strongly suggest a memory effect: The temporal or dynamic memory of the resting state is carried over to the task state as otherwise the latter could not be modulated by the former. Given that such temporal memory effect in terms of rest-task modulation was related to the self-non-self differentiation, one would strongly assume it to be self-specific.

How does such self-specific temporal memory of the resting state affect the task states? This was addressed by Kolvoort et al. (2020) in an EEG study on self. They measured resting state in EEG and conducted a psychological self-task where subjects were required to associate self- and non-self-specific stimuli across different time delays (from 200ms to 1,400ms). They demonstrate that the self-specific effects in terms of accuracy was preserved across all temporal delays with intersubject variation. That, in turn, was related to the resting state PLE: the higher the resting state PLE, that is, the stronger the slower frequencies relative to the faster ones, the stronger the self-specific effect was preserved across the different time delays on the psychological level. This suggests that temporal integration of different timescales as indexed by temporal memory may be key in mediating the co-occurrence temporal stability and flexibility of the self.

Together, these findings suggest that the self is intrinsically dynamic in that it integrates and combines temporal continuity and discontinuity across different timescales, that is, in a scale-free way. The data show that the brain’s degree of scale-freeness is key in mediating the self which, psychologically, is manifest in the link of temporal continuity and discontinuity. Since temporal discontinuity and continuity concern different timescales, that is, fast and slow, one can also speak of scale-freeness on the psychological level of self (which remains to be demonstrated empirically, though).

More generally, scale-freeness may be shared by both brain and self as their “common currency.” This points to the importance of (1) conceiving the brain in terms of dynamic, that is, scale-freeness of neural activity and (2) taking into view the corresponding manifestation of dynamic in organizing the psyche in a temporal way, that is, the scale-freeness of self. Accordingly, the example of self strongly encourages the utility and validity of Spatiotemporal Neuroscience for providing the intimate (and necessary) connection of brain and psyche through topography and dynamic.

## Part Iv: “Project For a Spatiotemporal Neuroscience” – Extending Freud and Solms

### “Project for a Spatiotemporal Neuroscience” – Complementing and Extending Freud

**Figure 7:**
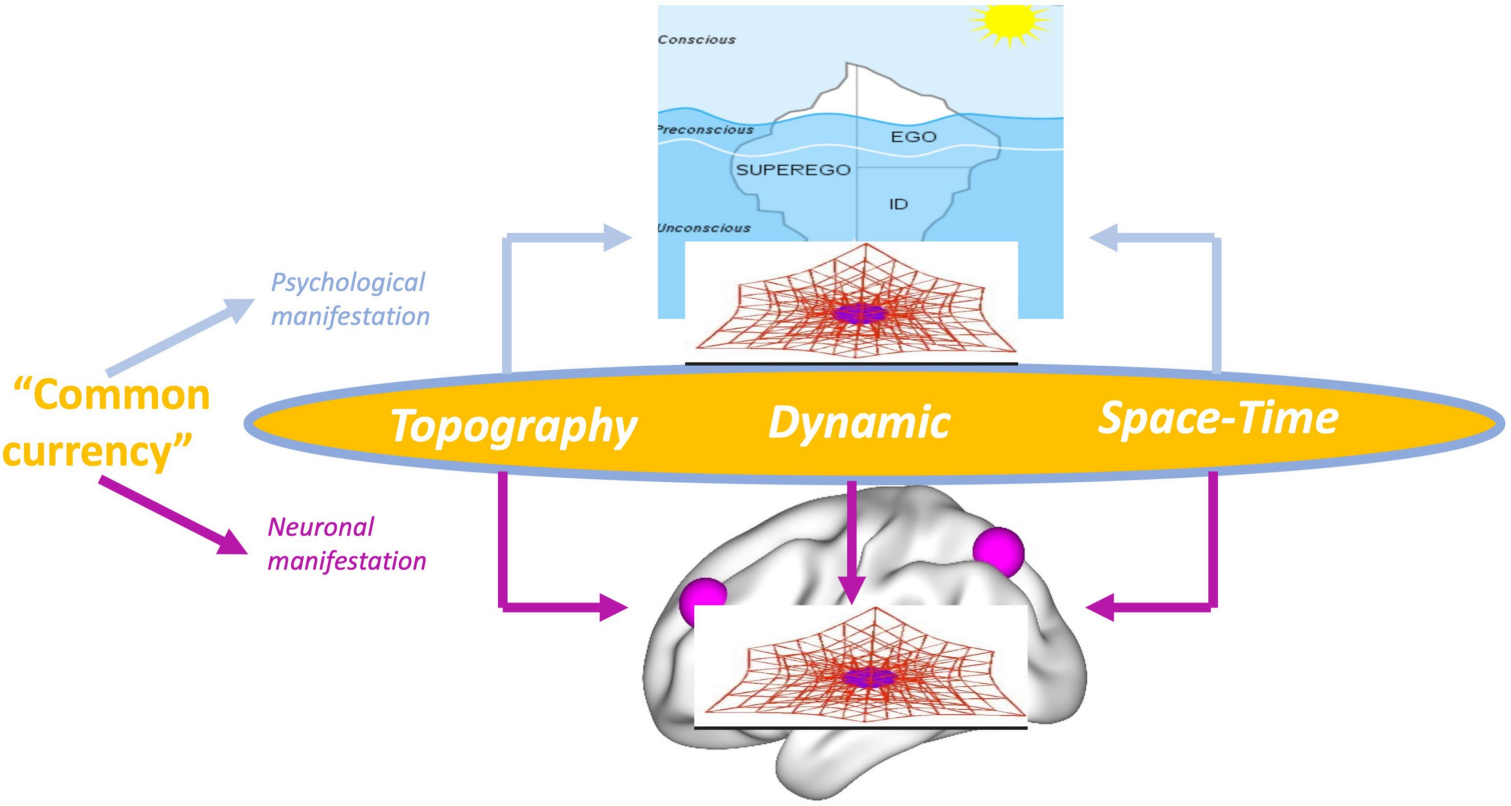
Shared features as “common currency” (middle) of brain and (lower) and psyche (upper).

After having provided empirical support for Spatiotemporal Neuroscience, we are now ready to provide an answer to Freud’s original quest for an intimate link of psyche and brain as developed in his “Project for a Scientific Psychology.” Following Freud’s view of the psyche, Spatiotemporal Neuroscience considers the brain’s neural activity as topographic, dynamic, and essentially spatiotemporal. Hence, spatial topographic and temporal dynamic features provide the features that are shared by the brain’s neural activity and the psyche’s psychodynamic features, their “common currency” (Figure 7).

Taken in a nutshell, Spatiotemporal Neuroscience provides the missing link of brain and psyche which remained elusive to Freud at his time. We therefore speak of the need for a “Project for a Spatiotemporal Neuroscience.” What do we mean by “Project for a Spatiotemporal Neuroscience” and how does it stand in relation to Freud’s original “Project for a Scientific Psychology”?

The “Project for a Spatiotemporal Neuroscience” aims to develop the kind of neuroscience that, by establishing a temporal dynamic and spatial topographic view of the brain and its various functions, allows for their intimate connection with the psyche’s psychodynamic features (see orange arrow in Figure 2). This complements and extends Freud’s original Project which, due to the lack of neuroscientific research at its time, could not conceive the dynamic and topography of the brain. Accordingly, the “Project for a Spatiotemporal Neuroscience” provides Freud with the kind of neuroscience that allows him to intimately link his view of the psyche to the brain and thus to complement his original project.

### “Project for a Spatiotemporal Neuroscience” II – Empirical Convergence With Solms’ “(New) Project for a Scientific Psychology”

How does our “Project for a Spatiotemporal Neuroscience” stand in relation to the recently proposed “(New) Project for a Scientific Psychology” by Mark Solms (Solms, 2020)? Mark Solms recently proposed a “New Scientific Psychology” (Solms, 2020, 2021) where he casts Freud’s original “Scientific Psychology” in the terms of free energy and predictive coding. He uses the physical-biological framework of affective neuroscience (AF, Panksepp, 1998), free energy principle (FEP) and predictive coding (PC) to account for psychodynamic concepts like memory, primary and secondary processes, cathexis, dreams, and the ego as basic structure or organization. Following Freud’s “Scientific Psychology,” he uses the original text as template for reformulating it in terms of Friston’s FEP coupled with the Affective Neuroscience by Panksepp, relying particularly on what Panksepp call the primal “SELF” where the role of the PAG and the brain stem is central for both *“the terminus of every affect circuit and the genesis of every newly felt affect”* (Solms, 2020, p.10).

How does Solms’ Project of a “New scientific Psychology” stand in relation to the here proposed “Project for a Spatiotemporal Neuroscience”? First and foremost, both are not exclusive but compatible. There is plenty of convergence between Panksepp’s AN, Friston’s FEP coupled with PC on the one hand and the spatiotemporal approach to the brain in terms of Spatiotemporal Neuroscience. His “(New) Project for a Scientific Psychology” thus converges with our “Project for a Spatiotemporal Neuroscience.”

Prediction and free energy are driven by a deeper layer of the brain’s temporal dynamics, that is, deep temporal models (Kiebel et al., 2008; Friston et al., 2017). Deeper layer is not here understood in terms of time and space scales but in terms of a deeper organizational and structuring principle that holds across all other subsequent layers as well as across all time–space scales. Spatiotemporal neuroscience may thus provide the temporal (and spatial topographic) underpinnings driving PC as we see in the case of the self. The same holds analogously in the case of FEP that has been demonstrated to be scale-free and that operated at multiple and nested spatial scale and timescale (Friston et al., 2015): These represent the intrinsic temporal dynamic and spatial topographical features of FEP. In other words, the spatiotemporal, that is, dynamic and topographic configurations in the matching of brain and environment are key in mediating the degree of free energy, that is, FEP. Accordingly, both FEP and PC may be driven, on a holistic and more fundamental level, by dynamic and topography. Spatiotemporal Neuroscience thus provides a deeper more holistic and comprehensive empirical layer of the brain that can integrate and make us better understand how the brain can yield PC and FEP. In this context, we tried to explicate a deeper layer in FEP and PC that drives and organizes both but is not yet by itself explicated as such. A similar relation can be found between Spatiotemporal Neuroscience and Panksepp’s AN. Also in this case, Spatiotemporal Neuroscience provides the dynamic and the topography that organize and structure the generation of affects and feelings. Again our approach is not exclusive but rather holistic and comprehensive in its’ own purpose to provides the topographical and dynamic ground on which the different functions and manifestation of the brain and psyche are generated. Hence, our “Project for a Spatiotemporal Neuroscience” empirically converges with and complements Solms’ “(New) Project for a Scientific Psychology.”

### “Project for a Spatiotemporal Neuroscience” III – Conceptual Extension of Solms’ “(New) Project for a Scientific Psychology”

Do we need both FEP/PC and Spatiotemporal Neuroscience? Or is one sufficient to explain the psyche? FEP/PC explain and mathematically formulate brilliantly the physical-biological features of the brain as both FEP and PC strongly borrow from physics and biology. However, that leaves open in both FEP/PC and Solms how the brain’s states are connected to and, ultimately, can transform into psychical or mental states. Let us highlight this point.

We are encountering theoretical and empirical questions in our aim to intimately connect brain and psyche: what provides the necessary condition or intrinsic feature of the transition and connection from brain to psyche? Why and how does the brain’s neural activity transform into psychic activity with its various functions (affective, social, cognitive, etc.) shaped by PC/FEP? Necessary connection (as theoretical concept) and transformation (as empirical concept) mean here that if the neuronal state appears in a particular way, it cannot avoid being associated with or entailing the presence of a particular psychical or mental state. We are thus encountering a “gap of contingency” between brain and psyche something that, in the specific instance of consciousness, has also described as “hard problem” in philosophy (Chalmers, 1996).

How can we close the “gap of contingency” between brain and psyche? This is the moment where Spatiotemporal Neuroscience, together with the assumption of “common currency,” comes in. Brain and psyche share spatial topographic and temporal dynamic features as their “common currency” which underlie and shape PC and FEP and subsequently the respective affective and cognitive functions. This, as detailed in Northoff (2018), provides an intrinsic or necessary *a posteriori* connection of brain and psyche. The “gap of contingency” can consequently be closed and, even stronger, be resolved by Spatiotemporal Neuroscience through its assumption of spatial topography and temporal dynamic providing the “common currency” of brain and psyche.

**Figure 8:**
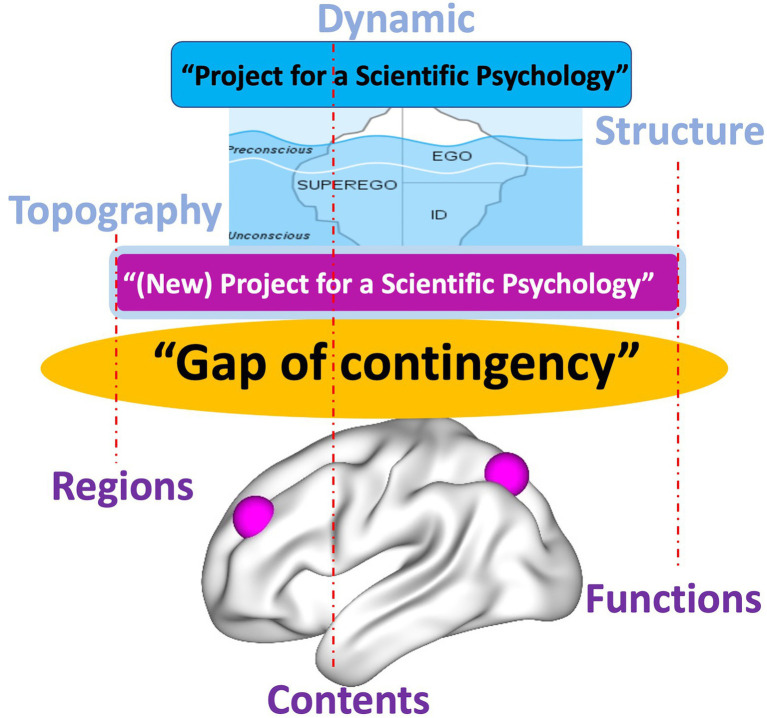
Freud’s “Project for a Scientific Psychology” and Solms’ “(New) Project for a Scientific Psychology” leave open the “Gap of contingency” (red dotted lines indicating insufficient, i.e., contingent connection) due to discrepant models of brain and psyche.

This carries major implications for the relationship of our “Project for a Spatiotemporal Neuroscience” to Solms’ “(New) Project for a Scientific Psychology.” By providing analogous views of brain and psyche in terms of topography, dynamic, and spatiotemporality, the “Project for a Spatiotemporal Neuroscience” bridges and resolves the “gap of contingency” of brain and psyche. Since the “gap of contingency” is still present in Friston’s concepts of FEP and PC, Solms’ “(New) Project for a Scientific Psychology” cannot avoid this gap either (Figure 8).

**Figure 9:**
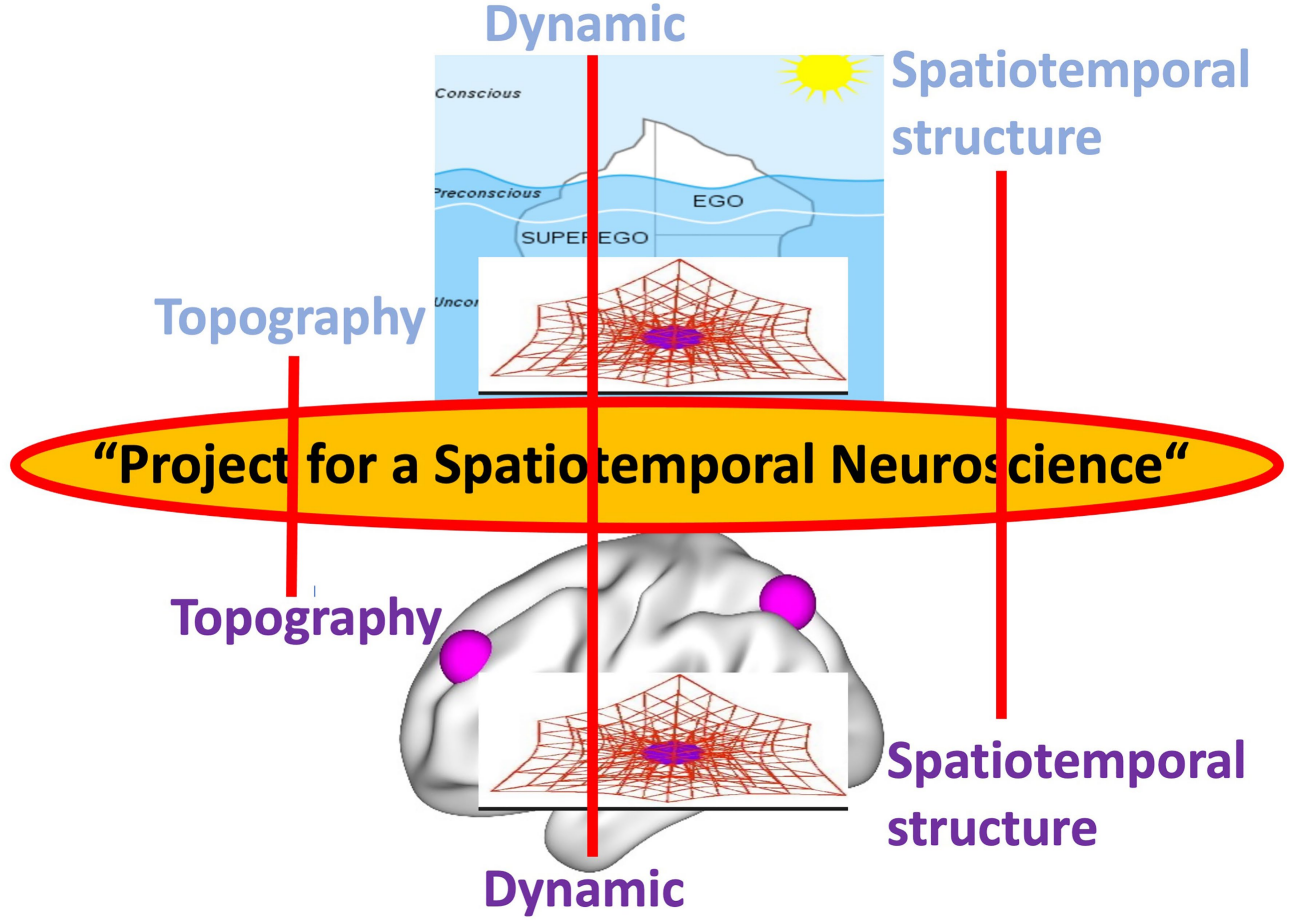
Shared features as “common currency” (middle) of brain and (lower) and psyche (upper).

This is the moment where the “(New) Project for a Scientific Psychology” may want to turn to our “Project for a Spatiotemporal Neuroscience”: the latter’s focus on the brain’s topography and dynamic providing the shared feature or “common currency” with the psyche can close the “gap of contingency” in Solms’ “(New) Project for a Scientific Psychology.” Taken in this sense, the “Project for a Spatiotemporal Neuroscience” conceptually extends the “(New) Project for a Scientific Psychology” by providing a more intimate, that is, necessary *a posteriori* (Northoff, 2018) connection of brain and psyche (Figure 9).

Closing the “gap of contingency” of brain and psyche is not only of theoretical-conceptual importance but also in a very practical sense. The “Project for a Spatiotemporal Neuroscience” allows us to develop a novel form of psychodynamic psychotherapy, namely, Spatiotemporal Psychotherapy. Although it remains to be fully explicated, we at least want to provide some initial hints about such temporally and spatially based psychotherapy.

### Spatiotemporal Psychotherapy I – Spatial and Temporal Integration of Self Through the Therapist

What is the goal of psychotherapy? In our neuroscientifically informed view, the goal of psychotherapy is (1) to reverse maladaptive topographic-dynamic re-organization of brain and (2) to establish a more adaptive and stable spatiotemporal nestedness of brain and self thereby re-establishing a proper nested hierarchy of self. This process, in accordance with contemporary psychoanalysis, might serve to re-establish the subjective sense of integrity, coherence, and continuity of self over time and space, similar to what has been described by Philip Bromberg: “health is the ability to stand in the spaces between realities without losing any of them – the capacity to feel like one self while being many” (Bromberg, 1996, p. 166).

Psychotherapeutically, this means that we may need to operates at the subjects’ level of perception (or experience) of time, i.e., dynamics, and space, i.e., topography as the building blocks of individuals self-states (and ultimately their brain’s temporal dynamic and spatial topographic structure) to remedy and heal their discrepancies, discontinuities, dis-integrity of the sense of self. In this context, we explicitly refer to contemporary psychoanalysis of self and relatedness (i.e., object relations) leaving beyond classical concept of psychoanalysis, such as drives, conflicts, and defense mechanisms. Our target is here to focus on the sense of self and its intrinsic features. Our aim is to neuroscientifically inform psychotherapy and expand our knowledge on the self and its intrinsic features at neuro-psychodynamic level. At the current stage, our model here does not aim to change or provides new therapeutic techniques; nevertheless, spatiotemporal psychotherapy provides a more comprehensive and neuroscientific informed framework that might be useful for therapists.

For instance, the therapist may need to operate at building blocks of consciousness and unconscious processing through spatial topographic and temporal dynamic means: the therapist needs to connect (virtually or symbolically) her/his larger (spatial topographic and temporal dynamic) scales of her/his own exteroceptive and/or mental self to the more restricted of his client’s interoceptive self. Pragmatically, this means that operating in the dual relational field, the therapist must operate in the transferential-contertransferential matrix using the “common currency” of time and space as the cardinal points to note and *work through* the moments of rupture of the sense of self and its intrinsic features.

This analytical dance in the transitional space and time of the real and the virtual relationship between the two subjects made by continuous “ruptures and repairs” provides the client with the opportunity to integrate and nest her/his own more restricted spatiotemporal scales of her/his interoceptive self in a virtual, that is, interpersonal way into the larger ones of her/his therapist. That, in turn, will allow the client to process the traumatic input relationships in a non-threatening and non-disrupting way for her/his own self without becoming fragmented and loosing the access to one’s interoceptive self. The traumatic input relationships associated with the own interoceptive self are now integrated and nested virtually (or symbolically) within the therapists’ larger spatiotemporal scales (of the therapist’s exteroceptive and mental self).

Accordingly, the therapeutic aim here is to spatially and temporally re-integrate the different layers of self: that serves the purpose to connect the different layers of self such that they can become conscious together rather than being split off and isolated into the dynamic unconscious (as in dissociation). Dissociation here operates in terms of lack of integration between the different layers of the self and seems to be mediated by the lack of connectivity (thus integrative function) in the right anterior insula with the rest of the brain at different spatiotemporal scales (Scalabrini et al., 2020b) accompanied by the loss of first-person perspective. Consequently, healing the self means to re-establish the sense of self-continuity beyond the dissociation of its trauma. This is possible by re-stablishing and/or re-organizing the topography and dynamic of the nested hierarchy of both self and its brain through spatial and temporal means – this amounts to what we here describe as “Spatiotemporal Psychotherapy.”

### Spatiotemporal Psychotherapy II – Timing, Spatialness, Dynamic, and Shared Time–Space

What is Spatiotemporal Psychotherapy? Spatiotemporal psychotherapy consists in modulating the individual’s subjectively perceived (consciously and unconsciously) time-scale and space-scale on both neural and psychological levels. This process calls into account the role of the therapist that here works at the edges of different affectives and self-states characterized by their respective time-scale and space-scale. The primary purpose of the therapist is to reach and integrate their clients’ dissociated spatiotemporal layers of self with their respective affects and thoughts (this is consistent with the work on different traumatic levels that has been clinically described by Mucci, 2013, 2018; Mucci and Scalabrini, 2021).

The primary means of such spatiotemporal psychotherapy are thus spatial and temporal in both intra-personal experience/perception and interpersonal transference. This targets the most basic and fundamental layers of existence (Scalabrini et al., 2020c), the spatiotemporal coordinates that tie together different people like therapist and client while, at the same time, being most vulnerable to traumatic events and influences. Importantly, the main therapeutic direction of client-therapist interaction is from their shared inter-personal space and time to the intra-personal experiences/perceptions of the client (and those of the therapist).

How does Spatiotemporal Psychotherapy work? For instance, the therapist may provide more stable, regular, and continuous mixture of slow and fast timescales trying to be “sufficiently” aligned with the patient in the analytic dance. This process aim to regularize, stabilize, and make the temporal dynamic flow of the client’s neural and psychic activity more continuous. While at the same time, this will allow integrating temporal discontinuity and change as related to traumata. This, as we hypothesize, should complement and mirror the client’s self-state increasing these subjects’ arousal level modulating their affect and emotion as well as their thought dynamic (Rostami et al., 2021). Hence, timing, spatialness, and temporal dynamic within the interaction of client and therapist will be key in such psychotherapeutic regulatory approach.

A psychotherapy that is interpersonally attuned in time and aligned in space might provide a more comprehensive, basic, and extensive operating field that also embed and contains affective, social, cognitive functions within a larger more comprehensive context. Here, we suggest the therapists to work using these spatiotemporal coordinates beyond the contents and the narratives of the patients. The shared time and space between therapist and client might here be seen as an operating commonly shared interpersonal spatiotemporal field, which makes possible the re-organization and transformation of the client’s intra-personal nested hierarchy of self through its spatiotemporal manifestation within her/his brain (See Spagnolo and Northoff, 2021).

**Figure 10:**
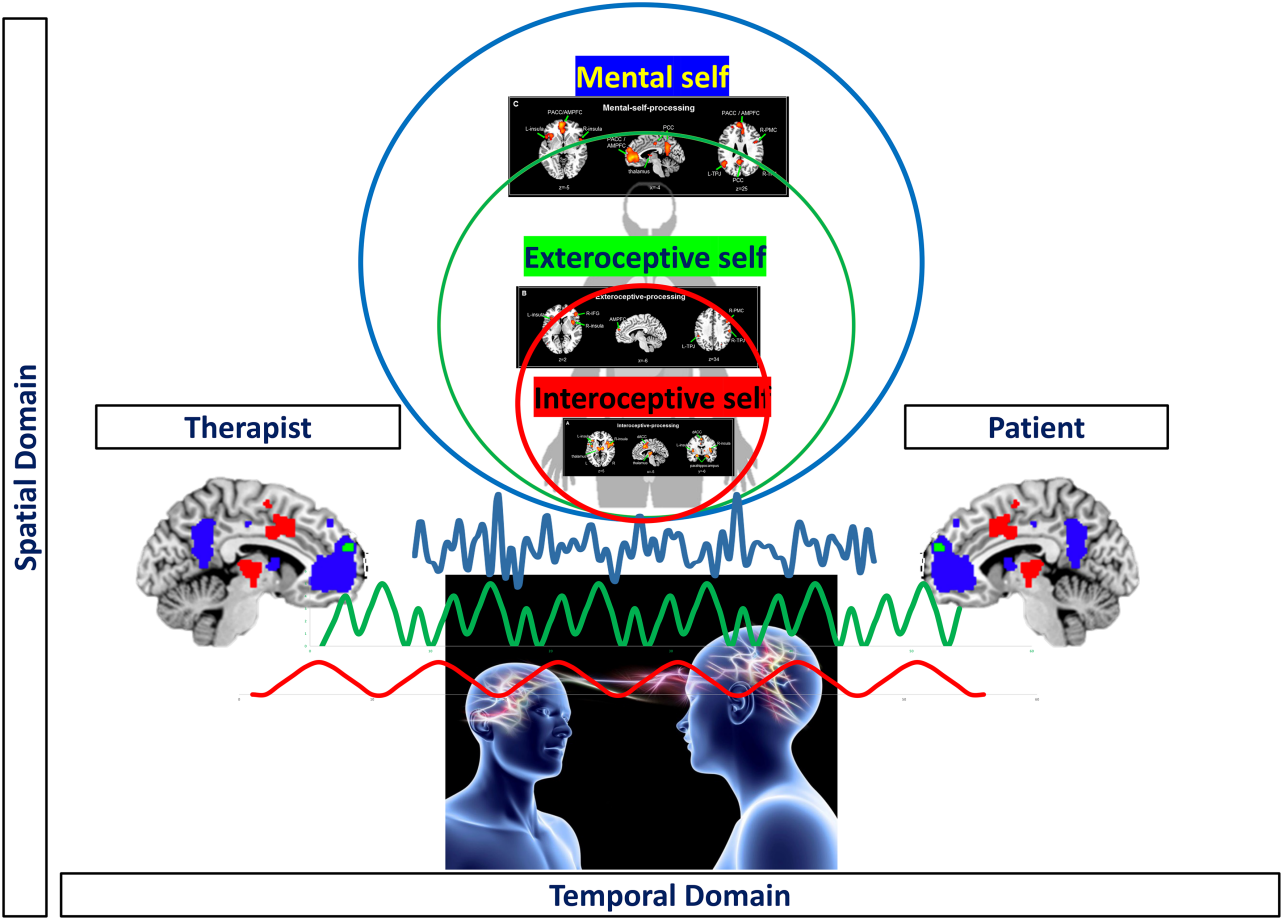
Visual representation of spatiotemporal psychotherapy.

In case of very severe psychiatric patients, one could also complement such temporo-spatial psychotherapy by brain-based intervention operating on the basis of the brain’s spatiotemporal features. For instance, transcranial magnetic stimulation may, if stimulating in the “right” frequency, can foster and facilitate slow-fast temporal integration on the neuronal level of, for instance, the default-mode network (DMN) in order to help the client to remit from dissociating her/his own mental self and to enlarge its spatial and temporal scales beyond those related to its “traumatic shrinking.” That, in turn, provides the ground for the more virtual or symbolic work with the therapist to re-order, re-integrate, and re-nest the client’s mental self within her/his own intero- and exteroceptive self (Figure 10).

## Conclusion – “Project For a Spatiotemporal Neuroscience”

### Mismatch of Brain and Psyche – Adapting the Model of Brain

Freud searched for the scientific basis of the psyche in the brain. He deemed his “Project for a Scientific Psychology” a failure, though, as he was not able to intimately the psyche’s topography, dynamic, and spatiotemporal features to corresponding features in the brain. Having more insight in our times, current neuropsychoanalysis aims to trace the psyche’s topography, dynamic, and spatiotemporality to the brain’s affective and cognitive (and social and cultural) functions including their predictive coding and free energy. However, we argue that, despite all progress, such approach does not yet fully complete the Freudian’ quest: It does not provide the kind of intimate, that is, necessary connection of brain and psyche that allows closing their “gap of contingency” which Freud deemed necessary to complete his project.

Specifically, the current neuropsychoanalytic approach suffers from a mismatch in its presupposed models of brain and psyche. Following Freud and others, the psyche is characterized by dynamic, topography, and spatiotemporality. That is related to a model of the brain in Cognitive, Social and Affective Neuroscience where the brain is conceived as static (rather than dynamic), modular (rather than topographic), and non-spatiotemporal (rather than spatiotemporal). This amounts to a mismatch between the models of psyche and brain, though. That, in turn, prevents us from taking into view the intimate or necessary brain-psyche connection one requires to properly complete Freud’s “Project for a Scientific Psychology.”

How can we adapt our model of brain to the model of psyche Freud and others envision in psychoanalysis? For that, we have to take into view the brain’s intrinsic spatial topography and temporal dynamics. This leads us to Spatiotemporal Neuroscience (Northoff et al., 2020a,b). Unlike its cognitive, affective, and social siblings, Spatiotemporal Neuroscience conceives the brain’s neural activity primarily in terms of topography, dynamic, and spatiotemporality which, in turn, structure and organize various functions including their predictive coding and free energy. Accordingly, conceiving brain and psyche in analogous ways, Spatiotemporal Neuroscience allows to take into view their intimate or necessary connection through shared features (“common currency”), that is, topography, dynamic, and spatiotemporality.

### “Project for a Spatiotemporal Neuroscience” – Complementing Freud and Solms

The introduction of such spatiotemporal model of the brain by Spatiotemporal neuroscience carries major implications for neuropsychoanalysis. Freud characterized the psyche by dynamic, topography, and spatiotemporality but was missing a corresponding model of the brain – his “Project for a Scientific Psychology” was thus doomed to failure. In a more recent brave attempt, Mark Solms aims to provide the missing pieces by proposing a “(New) Project for a Scientific Psychology” (Solms, 2020, 2021). By reverting to predictive coding and free energy, he provides some of the missing pieces of the puzzle but nevertheless leaves open the intimate or necessary connection of brain and psyche as he still relies largely on (the model of the brain provided by) Affective, Cognitive, and Social Neuroscience.

This is the moment where our proposed “Project for a Spatiotemporal Neuroscience” comes in. Spatiotemporal Neuroscience provides a model of the brain that is more or less analogous to Freud’s view of the psyche. That, in turn, makes it possible to take into view topography, dynamic, and spatiotemporality as the shared features of brain and psyche, their “common currency” (Northoff et al., 2020a,b). This closes the theoretical, conceptual, and empirical gap between brain and psyche, the “gap of contingency,” which both Freud and Solms did not overcome.

In conclusion, the “Project for a Spatiotemporal Neuroscience” complements Freud’s “Project for a Scientific Psychology” on theoretical grounds. At the same time, it converges empirically with and extends conceptually beyond Solms’ “(New) Project for a Scientific Psychology.” While practically, the “Project for a Spatiotemporal Neuroscience” lays the groundwork for a novel form of neuroscientific informed psychotherapy, namely Spatiotemporal Psychotherapy.

## Author Contributions

GN and AS wrote the article together. All authors contributed to the article and approved the submitted version.

## Funding

This project/research was supported by the Michael Smith Foundation Canada Research Chair, by the grant from the Ministry of Science and Technology of China, National Key RandD Program of China (2016YFC1306700) and from the European Union’s Horizon 2020 Framework Program for Research and Innovation under the Specific Grant Agreement No. 785907 (Human Brain Project SGA2), the ERANET grant, NFRF grant, and Team grant from uOMBRI to GN and by “Search for Excellence – UdA” (University G. d’Annunzio of Chieti Pescara) to AS for the project SYNC (The Self and its psYchological and Neuronal Correlates – Implications for the understanding and treatment of depression as a disorder of Self).

## Conflict of Interest

The authors declare that the research was conducted in the absence of any commercial or financial relationships that could be construed as a potential conflict of interest.

## Publisher’s Note

All claims expressed in this article are solely those of the authors and do not necessarily represent those of their affiliated organizations, or those of the publisher, the editors and the reviewers. Any product that may be evaluated in this article, or claim that may be made by its manufacturer, is not guaranteed or endorsed by the publisher.

## References

[ref1] Babo-Rebelo M. Buot A. Tallon-Baudry C. (2019). Neural responses to heartbeats distinguish self from other during imagination. NeuroImage 191, 10–20. doi: 10.1016/j.neuroimage.2019.02.012, PMID: 30738205 PMC6503945

[ref2] Babo-Rebelo M. Wolpert N. Adam C. Hasboun D. Tallon-Baudry C. (2016). Is the cardiac monitoring function related to the self in both the default network and right anterior insula? Philos. Trans. R. Soc. Lond. Ser. B Biol. Sci. 371:20160004. doi: 10.1098/rstb.2016.0004 28080963 PMC5062094

[ref3] Boeker H. (2018). “Psychoanalysis and neuroscience: The development of Neuropsychoanalysis,” in Neuropsychodynamic Psychiatry. eds. Boeker H. Hartwich P. Northoff G. (Cham: Springer), 19–48.

[ref4] Bromberg P. M. (1996). Standing in the spaces: The multiplicity of self and the psychoanalytic relationship. Cont. psychoanal. 32, 509–535. doi: 10.1080/00107530.1996.10746334

[ref5] Buzsaki G. (2006). Rhythms of the Brain. United Kingdom: Oxford University Press.

[ref6] Chalmers D. J. (1996). The Conscious Mind: In Search of a Fundamental Theory. United Kingdom: Oxford Paperbacks.

[ref7] Christoff K. Irving Z. C. Fox K. C. Spreng R. N. Andrews-Hanna J. R. (2016). Mind-wandering as spontaneous thought: a dynamic framework. Nat. Rev. Neurosci. 17, 718–731. doi: 10.1038/nrn.2016.113, PMID: 27654862

[ref8] Craig A. D. (2003). Interoception: the sense of the physiological condition of the body. Curr. Opin. Neurobiol. 13, 500–505. doi: 10.1016/S0959-4388(03)00090-4, PMID: 12965300

[ref9] Craig A. D. (2010). The sentient self. Brain Struct. Funct. 214, 563–577. doi: 10.1007/s00429-010-0248-y, PMID: 20512381

[ref10] Damasio A. (2010). Self Comes to Mind: Constructing the Conscious Brain. United States: Pantheon Books

[ref11] Edelman G. M. (1987). Neural Darwinism: The Theory of Neuronal Group Selection. United States: Basic books. 10.1126/science.240.4860.1802 17842436

[ref12] Edelman G. M. (1989). The Remembered Present: A Biological Theory of Consciousness. United States: Basic Books.

[ref13] Edelman G. M. (1992). Bright Air, Brilliant Fire: On the Matter of the Mind. United States: Basic books

[ref14] Freud S. (1895). Project for a Scientific Psychology. London: Hogarth Press.

[ref07] Freud S. (1900). The Interpretation of Dreams. S. E., 4 & 5. London: Hogarth.

[ref08] Freud S. (1915). The unconscious. S. E., 14, 166–204. London: Hogarth.

[ref15] Friston K. Levin M. Sengupta B. Pezzulo G. (2015). Knowing one's place: a free-energy approach to pattern regulation. J. R. Soc. Interface 12:20141383. doi: 10.1098/rsif.2014.1383, PMID: 25788538 PMC4387527

[ref16] Friston K. J. Parr T. de Vries B. (2017). The graphical brain: belief propagation and active inference. Netw Neurosci. 1, 381–414. doi: 10.1162/NETN_a_00018, PMID: 29417960 PMC5798592

[ref17] Gallagher S. (2005). Dynamic models of body schematic processes. Adv. Conscious. Res. 62, 233–250. doi: 10.1075/aicr.62.15gal

[ref18] Hasson U. Chen J. Honey C. J. (2015). Hierarchical process memory: memory as an integral component of information processing. Trends Cogn. Sci. 19, 304–313. doi: 10.1016/j.tics.2015.04.006, PMID: 25980649 PMC4457571

[ref19] He B. J. (2014). Scale-free brain activity: past, present, and future. Trends Cogn. Sci. 18, 480–487. doi: 10.1016/j.tics.2014.04.003, PMID: 24788139 PMC4149861

[ref20] He B. J. Zempel J. M. Snyder A. Z. Raichle M. E. (2010). The temporal structures and functional significance of scale-free brain activity. Neuron 66, 353–369. doi: 10.1016/j.neuron.2010.04.020, PMID: 20471349 PMC2878725

[ref21] Huang Z. Obara N. Davis H. H. 4th Pokorny J. Northoff G. (2016). The temporal structure of resting-state brain activity in the medial prefrontal cortex predicts self-consciousness. Neuropsychologia, 82, 161–170. doi: 10.1016/j.neuropsychologia.2016.01.025, PMID: 26805557

[ref22] Kernberg O. F. (1984). Severe Personality Disorders: Psychotherapeutic Strategies. New Haven, CT: Yale UP.

[ref23] Kiebel S. J. Daunizeau J. Friston K. J. (2008). A hierarchy of time-scales and the brain. PLoS Comput. Biol. 4:e1000209. doi: 10.1371/journal.pcbi.1000209, PMID: 19008936 PMC2568860

[ref24] Kolvoort I. R. Wainio-Theberge S. Wolff A. Northoff G. (2020). Temporal integration as “common currency” of brain and self-scale-free activity in resting-state EEG correlates with temporal delay effects on self-relatedness. Hum. Brain Mapp. 41, 4355–4374. doi: 10.1002/hbm.25129, PMID: 32697351 PMC7502844

[ref25] Leuzinger-Bohleber M. (2018). Finding the Body in the Mind: Embodied Memories, Trauma, and Depression. United Kingdom: Routledge.

[ref26] Linkenkaer-Hansen K. Nikouline V. V. Palva J. M. Ilmoniemi R. J. (2001). Long-range temporal correlations and scaling behavior in human brain oscillations. J. Neurosci. 21, 1370–1377. doi: 10.1523/JNEUROSCI.21-04-01370.2001, PMID: 11160408 PMC6762238

[ref27] Logothetis N. K. Murayama Y. Augath M. Steffen T. Werner J. Oeltermann A. (2009). How not to study spontaneous activity. NeuroImage 45, 1080–1089. doi: 10.1016/j.neuroimage.2009.01.010, PMID: 19344685

[ref28] Longo M. R. Tsakiris M. (2013). Merging second-person and first-person neuroscience. Behav. Brain Sci. 36:429. doi: 10.1017/S0140525X12001975, PMID: 23883758 PMC3772344

[ref29] MacLean P. D. (1990). The Triune Brain in Evolution: Role in Paleocerebral Functions. Germany: Springer Science and Business Media.

[ref30] Menon V. (2011). Large-scale brain networks and psychopathology: a unifying triple network model. Trends Cogn. Sci. 15, 483–506. doi: 10.1016/j.tics.2011.08.003, PMID: 21908230

[ref31] Mucci C. (2013). Beyond Individual and Collective Trauma: Intergenerational Transmission, Psychoanalytic Treatment, and the Dynamics of Forgiveness. United Kingdom: Routledge.

[ref32] Mucci C. (2018). Borderline Bodies: Affect Regulation Therapy for Personality Disorders (Norton Series on Interpersonal Neurobiology). United States: WW Norton and Company.

[ref33] Mucci C. (2019). Traumatization Through human agency: “embodied witnessing” is essential in the treatment of survivors. Am. J. Psychoanal. 79, 540–554. doi: 10.1057/s11231-019-09225-y, PMID: 31723219

[ref34] Mucci C. Scalabrini A. (2021). Traumatic effects beyond diagnosis: The impact of dissociation on the mind-body-brain system. Psychoanal. Psychol. doi: 10.1037/pap0000332 [Epub ahead of print]

[ref35] Northoff G. (2011). Neuropsychoanalysis in Practice: Brain, Self and Objects. United Kingdom: Oxford University Press.

[ref36] Northoff G. (2012a). Psychoanalysis and the brain - why did freud abandon neuroscience? Front. Psychol. 3:71. doi: 10.3389/fpsyg.2012.00071 22485098 PMC3317371

[ref37] Northoff G. (2012b). Immanuel Kant's mind and the brain's resting state. Trends Cogn. Sci. 16, 356–359. doi: 10.1016/j.tics.2012.06.001 22748399

[ref38] Northoff G. (2014a). Unlocking the Brain. Coding. Vol. 1. United Kingdom: Oxford University Press.

[ref39] Northoff G. (2014b). Unlocking the Brain. Consciousness. Vol. 2. United Kingdom: Oxford University Press

[ref40] Northoff G. (2016). Is the self a higher-order or fundamental function of the brain? The “basis model of self-specificity” and its encoding by the brain’s spontaneous activity. Cogn. Neurosci. 7, 203–222. doi: 10.1080/17588928.2015.1111868, PMID: 26505808

[ref41] Northoff G. (2017). Personal identity and cortical midline structure (CMS): do temporal features of CMS neural activity transform into “self-continuity”? Psychol. Inq. 28, 122–131. doi: 10.1080/1047840X.2017.1337396

[ref42] Northoff G. (2018). The Spontaneous Brain: From the Mind-Body to the World-Brain Problem. United States: MIT Press.

[ref020] Northoff G. Panksepp J. (2008). The trans-species concept of self and the subcortical–cortical midline system. Trends Cogn. Sci. 12, 259–264. doi: 10.1016/j.tics.2008.04.007, PMID: 18555737

[ref43] Northoff G. Bermpohl F. Schoeneich F. Boeker H. (2007). How does our brain constitute defense mechanisms? First-person neuroscience and psychoanalysis. Psychother. Psychosom. 76, 141–153. doi: 10.1159/000099841, PMID: 17426413

[ref44] Northoff G. Heinzel A. De Greck M. Bermpohl F. Dobrowolny H. Panksepp J. (2006). Self-referential processing in our brain—a meta-analysis of imaging studies on the self. NeuroImage 31, 440–457. doi: 10.1016/j.neuroimage.2005.12.002, PMID: 16466680

[ref45] Northoff G. Huang Z. (2017). How do the brain's time and space mediate consciousness and its different dimensions? Temporo-spatial theory of consciousness (TTC). Neurosci. Biobehav. Rev. 80, 630–645. doi: 10.1016/j.neubiorev.2017.07.013, PMID: 28760626

[ref46] Northoff G. Qin P. Feinberg T. E. (2011). Brain imaging of the self--conceptual, anatomical and methodological issues. Conscious. Cogn. 20, 52–63. doi: 10.1016/j.concog.2010.09.011, PMID: 20932778

[ref47] Northoff G. Wainio-Theberge S. Evers K. (2020a). Is temporo-spatial dynamics the “common currency” of brain and mind? In quest of “spatiotemporal neuroscience”. Phys Life Rev 33, 34–54. doi: 10.1016/j.plrev.2019.05.002 31221604

[ref48] Northoff G. Wainio-Theberge S. Evers K. (2020b). Spatiotemporal neuroscience - what is it and why we need it. Phys Life Rev 33, 78–87. doi: 10.1016/j.plrev.2020.06.005 32684435

[ref49] Panksepp J. (1998). The periconscious substrates of consciousness: affective states and the evolutionary origins of the self. J. Conscious. Stud. 5, 566–582.

[ref50] Panksepp J. (2012). What is an emotional feeling? Lessons about affective origins from cross-species neuroscience. Motiv. Emot. 36, 4–15. doi: 10.1007/s11031-011-9232-y

[ref51] Panksepp J. Biven L. (2012). The Archaeology of Mind: Neuroevolutionary Origins of Human Emotions (Norton Series on Interpersonal Neurobiology). United States: WW Norton and Company.

[ref52] Przyrembel M. Smallwood J. Pauen M. Singer T. (2012). Illuminating the dark matter of social neuroscience: considering the problem of social interaction from philosophical, psychological, and neuroscientific perspectives. Front. Hum. Neurosci. 6:190. doi: 10.3389/fnhum.2012.00190, PMID: 22737120 PMC3380416

[ref53] Qin P. Wang M. Northoff G. (2020). Linking bodily, environmental and mental states in the self-A three-level model based on a meta-analysis. Neurosci. Biobehav. Rev. 115, 77–95. doi: 10.1016/j.neubiorev.2020.05.004, PMID: 32492474

[ref54] Raichle M. E. (2009). A paradigm shift in functional brain imaging. J. Neurosci. 29, 12729–12734. doi: 10.1523/JNEUROSCI.4366-09.2009, PMID: 19828783 PMC6665302

[ref55] Raichle M. E. (2010). Two views of brain function. Trends Cogn. Sci. 14, 180–190. doi: 10.1016/j.tics.2010.01.008, PMID: 20206576

[ref56] Rostami S. Borjali A. Eskandari H. Rostami R. Northoff G. (2021). Novel approach to mind wandering in major depressive disorder and bipolar disorder patients: does the direction of thoughts matter? Int. J. Behav. Sci. 15, 66–72. doi: 10.30491/ijbs.2021.250747.1383

[ref57] Scalabrini A. Ebisch S. J. H. Huang Z. Di Plinio S. Perrucci M. G. Romani G. L.. (2019). Spontaneous brain activity predicts task-evoked activity During animate versus inanimate touch. Cereb. Cortex 29, 4628–4645. doi: 10.1093/cercor/bhy340, PMID: 30668664

[ref58] Scalabrini A. Huang Z. Mucci C. Perrucci M. G. Ferretti A. Fossati A.. (2017). How spontaneous brain activity and narcissistic features shape social interaction. Sci. Rep. 7:9986. doi: 10.1038/s41598-017-10389-9 28855682 PMC5577167

[ref59] Scalabrini A. Mucci C. Angeletti L. L. Northoff G. (2020c). The self and its world: a neuro-ecological and temporo-spatial account of existential fear. Clin. Neuropsychiatry 17, 46–58. doi: 10.36131/clinicalnpsych20200203 PMC8629082 34908967

[ref60] Scalabrini A. Mucci C. Esposito R. Damiani S. Northoff G. (2020b). Dissociation as a disorder of integration–On the footsteps of Pierre Janet. Prog. Neuro-Psychopharmacol. Biol. Psychiatry 101:109928. doi: 10.1016/j.pnpbp.2020.109928 32194203

[ref61] Scalabrini A. Mucci C. Northoff G. (2018). Is our self related to personality? A Neuropsychodynamic model. Front. Hum. Neurosci. 12:346. doi: 10.3389/fnhum.2018.00346 30337862 PMC6180150

[ref62] Scalabrini A. Vai B. Poletti S. Damiani S. Mucci C. Colombo C.. (2020a). All roads lead to the default-mode network—global source of DMN abnormalities in major depressive disorder. Neuropsychopharmacology 45, 2058–2069. doi: 10.1038/s41386-020-0785-x 32740651 PMC7547732

[ref090] Scalabrini A. Xu J. Northoff G. (2021). What COVID‐19 tells us about the self: The deep intersubjective and cultural layers of our brain. Psychiatry Clin. Neurosci. 75, 37–45. doi: 10.1111/pcn.13185 33305486

[ref63] Schacter D. L. Addis D. R. Hassabis D. Martin V. C. Spreng R. N. Szpunar K. K. (2012). The future of memory: remembering, imagining, and the brain. Neuron 76, 677–694. doi: 10.1016/j.neuron.2012.11.001, PMID: 23177955 PMC3815616

[ref64] Schilbach L. (2010). A second-person approach to other minds. Nat. Rev. Neurosci. 11:449. doi: 10.1038/nrn2805-c1, PMID: 20485366

[ref65] Seth A. K. (2015). Neural coding: rate and time codes work together. Curr. Biol. 25, R110–R113. doi: 10.1016/j.cub.2014.12.043, PMID: 25649819

[ref66] Smallwood J. Bernhardt B. C. Leech R. Bzdok D. Jefferies E. Margulies D. S. (2021). The default mode network in cognition: a topographical perspective. Nat. Rev. Neurosci., 22, 503–513. doi: 10.1038/s41583-021-00474-4 34226715

[ref67] Smallwood J. Schooler J. W. (2015). The science of mind wandering: empirically navigating the stream of consciousness. Annu. Rev. Psychol. 66, 487–518. doi: 10.1146/annurev-psych-010814-015331, PMID: 25293689

[ref68] Solms M. (2015). Reconsolidation: turning consciousness into memory. Behav. Brain Sci. 38:e24. doi: 10.1017/S0140525X14000296, PMID: 26050688

[ref69] Solms M. (2020). New project for a scientific psychology: general scheme. Neuropsychoanalysis 22, 5–35. doi: 10.1080/15294145.2020.1833361

[ref70] Solms M. (2021). The Hidden Spring: A Journey to the Source of Consciousness. United States: WW Norton and Company.

[ref71] Solms M. Friston K. (2018). How and why consciousness arises: some considerations from physics and physiology. J. Conscious. Stud. 25, 202–238.

[ref72] Spagnolo R. Northoff G. (2021). The Dynamic Self in Psychoanalysis. Neuroscientific Foundations and Clinical Cases. New York Routledge. doi: 10.4324/9781003221876

[ref73] Strachey J. (1961). Editor’s Introduction. Germany: Springer, 3–11.

[ref74] Tanabe S. Huang Z. Zhang J. Chen Y. Fogel S. Doyon J.. (2020). Altered global brain signal during physiologic, pharmacologic, and pathologic states of unconsciousness in humans and rats. Anesthesiology 132, 1392–1406. doi: 10.1097/ALN.0000000000003197, PMID: 32205548 PMC8218242

[ref75] Tsakiris M. (2017). The multisensory basis of the self: From body to identity to others. Q. J. Exp. Psychol. 70, 597–609. doi: 10.1080/17470218.2016.1181768 PMC5214748 27100132

[ref76] Wiest G. (2012). Neural and mental hierarchies. Front. Psychol. 3:516. doi: 10.3389/fpsyg.2012.00516 23189066 PMC3505872

[ref77] Wolff A. Di Giovanni D. A. Gómez-Pilar J. Nakao T. Huang Z. Longtin A.. (2019). The temporal signature of self: temporal measures of resting-state EEG predict self-consciousness. Hum. Brain Mapp. 40, 789–803. doi: 10.1002/hbm.24412 30288845 PMC6865612

[ref78] Yeshurun Y. Nguyen M. Hasson U. (2021). The default mode network: where the idiosyncratic self meets the shared social world. Nat. Rev. Neurosci. 22, 181–192. doi: 10.1038/s41583-020-00420-w, PMID: 33483717 PMC7959111

[ref79] Zhang J. Huang Z. Tumati S. Northoff G. (2020). Rest-task modulation of fMRI-derived global signal topography is mediated by transient coactivation patterns. PLoS Biol. 18:e3000733. doi: 10.1371/journal.pbio.3000733, PMID: 32649707 PMC7375654

